# An effective vaccine against influenza A virus based on the matrix protein 2 (M2)

**DOI:** 10.1016/j.vetmic.2024.110245

**Published:** 2024-09-14

**Authors:** Federico A. Zuckermann, Yelena V. Grinkova, Robert J. Husmann, Melissa Pires-Alves, Suzanna Storms, Wei-Yu Chen, Stephen G. Sligar

**Affiliations:** a Department of Pathobiology, College of Veterinary Medicine, University of Illinois Urbana-Champaign, Urbana, IL 61802, USA; b Department of Biochemistry, 505 South Goodwin Avenue, University of Illinois Urbana-Champaign, Urbana, IL 61801, USA

**Keywords:** Swine, Influenza A virus, Matrix protein 2, Vaccine, Nanodisc, Immunity, Nanoparticle

## Abstract

The ever-increasing antigenic diversity of the hemagglutinin (HA) of influenza A virus (IAV) poses a significant challenge for effective vaccine development. Notably, the matrix protein 2 (M2) is a highly conserved 97 amino acid long transmembrane tetrameric protein present in the envelope of IAV. More than 99 % of IAV strains circulating in American swine herds share the identical pandemic (pdm) isoform of M2, making it an ideal target antigen for a vaccine that could elicit broadly protective immunity. Here, using soluble nanoscale membrane assemblies termed nanodiscs (NDs), we designed this membrane mimetic nanostructures displaying full-length M2 in its natural transmembrane configuration (M2ND). Intramuscular (IM) immunization of swine with M2ND mixed with conventional emulsion adjuvant elicited M2-specific IgG antibodies in the serum that recognized influenza virions and M2-specific interferon-γ secreting cells present in the blood. Intranasal (IN) immunization with M2ND adjuvanted with a mycobacterial extract elicited M2-specific IgA in mucosal secretions that also recognized IAV. Immunization with an influenza whole inactivated virus (WIV) vaccine supplemented with a concurrent IM injection of M2ND mixed with an emulsion adjuvant increased the level of protective immunity afforded by the former against a challenge with an antigenically distinct H3N2 IAV, as exhibited by an enhanced elimination of virus from the lung. The lone IM administration of the M2ND vaccine mixed with an emulsion adjuvant provided measurable protection as evidenced by a >10-fold reduction or complete elimination of the challenge virus from the lung, but it did not diminish the viral load in nasal secretions nor the extent of pneumonia that ensued after the virus challenge. In contrast, an improved formulation of the M2ND vaccine that incorporated synthetic CpG oligodeoxynucleotides (CpG-ODN) in the nanostructures administered alone, via the IN and IM routes combined, provided a significant level of protective immunity against IAV as evidenced by a decreased viral load in both the upper and lower respiratory tracts and fully eliminated the occurrence of pneumonia in 89 % of the pigs immunized with this biologic. Notably, to be effective, the M2 protein must be displayed in the ND assemblies, as shown by the observation that simply mixing M2 with empty NDs incorporating CpG-ODN (eND-CpG-ODN) did not provide protective immunity. This novel M2-based vaccine offers great promise to help increase the breadth of protection afforded by conventional WIV vaccines against the diversity of IAV in circulation and, plausibly, as a broadly protective stand-alone biologic.

## 1. Introduction

The syndrome resulting from the infection of swine with influenza A virus (IAV) presents a major economic burden to the pork industry and is one of the top three diseases affecting pigs in all phases of pork production. Except for the 2009 H1N1 virus, influenza viruses that circulate in swine are very different from influenza viruses that commonly circulate in people. In recent years, the main flu viruses circulating in American swine farms are “triple-reassortant” H1N1, H1N2 and H3N2 lineage viruses (Vincent et al., 2017; Mancera Gracia et al., 2020; Vincent et al., 2020; Anderson et al., 2021). Effective vaccines are the cornerstone of defense against acute influenza virus infections. Experimental data indicates that the protection provided to swine by conventional whole inactivated virus (WIV) influenza vaccines is limited due in part to the constant antigenic drift of the hemagglutinin (HA) and neuraminidase (NA) envelope proteins and the need for antigenic match between the vaccine and the challenge virus (Vincent et al., 2017; Everett et al., 2019; Mancera Gracia et al., 2020; Salvesen and Whitelaw, 2021). Antigenic drift is caused by point mutations and is defined as the minor gradual antigenic changes in the HA or NA protein. HA antigenic drift variants result from the positive selection of spontaneously arising mutants by neutralizing antibodies i.e., antibody escape mutants. The constantly increasing diversity of the HA antigen presents a significant challenge for effective vaccine development (Vincent et al., 2017; Ma, 2020). The effectiveness of the seasonal influenza vaccine for humans ranges between 10 % and 60 %. However, even when well matched, the vaccine can lose efficacy. Between the time flu viruses are chosen as vaccine strains and when the flu vaccines are delivered, the circulation of influenza viruses in a particular season could have changed in ways that impact the vaccine’s effectiveness (CDC Vaccine Effectiveness). Commercially available IAV vaccines for swine are primarily adjuvanted whole inactivated virus (WIV) vaccines delivered intramuscularly (IM). These vaccines offer limited efficacy against intrasubtypic antigenic variants and heterosubtypic strains (Ma, 2020; Ma et al., 2023). This lack of antigenic match in commercial WIV vaccines in swine worsens due to the time needed for regulatory approval of veterinary vaccine products (Anderson et al., 2021). In addition, following licensure, there is no continued evaluation by regulatory authorities on efficacy of licensed swine vaccines in the field (Vincent et al., 2017).

The failure of currently available vaccines for human use to protect against HA antigen drifted seasonal influenza viruses or from antigenically novel pandemic viruses has driven the field toward efforts to develop a broadly protective “universal influenza vaccine" that can overcome the phenomena of HA antigenic changes (Erbelding et al., 2018). The matrix protein 2 (M2) is a conserved protein present in the envelope of IAV; hence, it is an ideal target antigen for the development of a broadly protective vaccine (Kolpe et al., 2017; Mezhenskaya et al., 2019; Saelens, 2019). Of the three IAV envelope proteins, namely HA, NA, and M2, the latter stimulates the weakest immune response (Feng et al., 2006; Kolpe et al., 2017), which is likely due to its scarcity in the virus envelope (Zebedee and Lamb, 1988). M2 is a 97 amino acid long transmembrane protein that forms a tetramer in the virus envelope and acts as a viroporin. M2 consists of an intravirion C-terminal domain (positions 47–97), a transmembrane domain (positions 25–46), and a N-terminal ectodomain (positions 1–24). Due to the high degree of conservation, the ectodomain of M2 (M2e) is a leading antigen candidate being tested for the development of a “universal” influenza vaccine for humans (Saelens, 2019). Since M2 is expressed in the viral envelope as a tetramer, a major technical issue for developing a M2e-based vaccine has been the difficulty of expressing M2e in its tetrameric configuration. Several M2e expression platforms and adjuvants have been investigated to express M2e as a tetramer and to be sufficiently immunogenic to function as an effective vaccine in mice (Wang et al., 2014; Zhang et al., 2014; Deng et al., 2015). However, despite >25 years of effort, no M2e-based vaccine for IAV is on the market.

It is notable that beginning in 2009, the pandemic M (pM) gene segment derived from the A/(H1N1) pdm09 virus, which caused the influenza pandemic in 2009 (Garten et al., 2009), has gradually displaced the presence of the triple reassortment M gene segment in influenza A viruses circulating in swine (Anderson et al., 2013). Currently, >99 % of the IAV in swine passively monitored through a U.S. Department of Agriculture (USDA) surveillance system express the pM gene segment (Arendsee et al., 2021). Hence, given that >99 % of IAV strains circulating in American swine herds express the pandemic (pdm) isoform of M2 (pM2), we undertook the effort to develop a broadly protective vaccine for swine based on pM2. However, unlike all other versions of M2-based vaccines that have been attempted, we investigated the notion of using the entire M2 protein as the immunogen displayed in its natural transmembrane configuration in nanostructures called nanodiscs (NDs). NDs are soluble and stable discoid shaped nanoscale structures consisting of a discrete lipid bilayer bound by two amphipathic membrane scaffold proteins. Because NDs epitaxially display both sides of the membrane’s lipid bilayer, they provide a native-like environment for transmembrane proteins to be displayed in their natural configuration. For this reason, NDs have become an important tool for studying the structural biology of membrane proteins (Bayburt et al., 2002; Denisov and Sligar, 2017; Sligar and Denisov, 2021). NDs consist of monodisperse discoidal particles 5.5 nm high and nominally 10 nm in diameter formed via a self-assembly process containing two copies of an alpha-helical, amphipathic protein, termed membrane scaffold protein (MSP). The phospholipids associate as a bilayer domain, while two molecules of MSP wrap around the edges of the discoidal structure in a belt-like configuration; one MSP covers the hydrophobic alkyl chains of each leaflet. Here, we report studies testing the immunogenicity and protective efficacy of a vaccine based M2 displayed in ND (M2ND). Initial experiments revealed that intramuscular immunization of swine with M2NDs mixed with a conventional water-oil-water (w/o/w) adjuvant elicited M2-specific IgG antibodies in serum capable of recognizing IAV virions, as well as M2-specific interferon-gamma secreting cells (IFN-γ-SC). Experimentation aimed at testing the ability of the M2NDs to increase the protective immunity afforded by whole inactivated virus (WIV) vaccines against IAV expressing an antigenically distinct HA or to confer protective immunity on their own led to the development of a vaccine formulation that incorporated synthetic CpG oligodeoxynucleotides (CpG-ODN) in the M2ND. The immunization of swine with M2ND-CpG-ODN provided a significant level of protective immunity against IAV. Notably, to be effective, the M2 protein must be displayed in the ND assemblies. This was shown by the observation that by simply mixing M2 with empty ND (eND) that incorporated CpG-ODN (eND-CpG-ODN), did not provide protective immunity. This novel M2-based vaccine offers great promise to help increase the breadth of protection afforded by conventional WIV vaccines against the diversity of IAV in circulation and, plausibly, as a broadly protective stand-alone biologic against IAV.

## 2. Materials and Methods

### 2.1. Viruses and inactivated IAV vaccines

The influenza A viruses used to either prepare whole inactivated virus (WIV) vaccines or to challenge pigs were: A/swine/NY/A01104005/2011 H3N2 (NY/11), which belongs to the H3 clade IV-A; and A/swine/Minnesota/A01668936/2016 H3N2 (MN/16), which belongs to the H3 Clade IV-B. These two H3N2 viruses were chosen because their H3 hemagglutinin subtype express disparate H3 antigenic motifs (Bolton et al., 2019). While the NY/11 virus, which expresses the antigenic motif NYNNYK that belongs to the H3 Red antigenic cluster, the MN/16 virus expresses the antigenic motif KYNNYK that belongs to the H3 Green antigenic cluster (Bolton et al., 2019). Both viruses were obtained from the National Veterinary Services Laboratory (NVSL) and derived from the same 1998 introduction/lineage; both have the same pandemic (pdm)-Matrix (M) gene and, therefore, express the same pdm M2 isoform. The viruses were propagated in Madin-Darby canine kidney (MDCK) cells, and clarified supernatants of virus-infected cultures were titrated using a plaque forming unit (PFU) assay as previously described (Baker, 2022) and are reported as PFU/ml. The WIV vaccines were prepared using UV-irradiated virus with the addition of a commercial oil-in-water (o/w) adjuvant (Emulsigen D, MVP Laboratories) at 20 % (v:v). A vaccine dose consisted of 2 ml containing 250 hemagglutination units (HAU) of either MN/16 or NY/11 virus as previously described (Vincent et al., 2010). Alternatively, where indicated, the commercial tetravalent swine influenza vaccine FluSure XP (Zoetis) was used following the manufacturer’s recommendations.

### 2.2. Experimental design / Animal immunization and challenge studies

All pigs enrolled in the four studies were obtained at 3–4 weeks of age from the UIUC Swine Research Farm free of IAV and porcine reproductive and respiratory syndrome virus. A total of four studies involving pigs were performed. A summary of the experimental design of these studies and the corresponding data is presented in Table 1.

### 2.3. Pig study 1

The first experiment assessed the immunogenicity of M2NDs. Sixteen pigs were divided into four groups (each group had n=4). Pigs in group 1 received intramuscular (IM) injections formulated in a 2 ml volume, with the M2ND (45 mg per dose) mixed 1:1 with the commercial water-in-oil-in-water (w/o/w) emulsion adjuvant ISA 206 (Seppic). Pigs in group 2 received intranasal (IN) immunizations of M2ND (45 mg per dose) mixed with a whole cell lysate of Mycobacteria smegmatis (100 mg per dose) administered using an Ideal Prima Vaccinator fitted with an Ideal^®^ Prima^®^ Mist Sprayer (Neogen, Lansing, MI). Lysates of *M. smegmatis* have been shown to serve as a mucosal adjuvant for the IN immunization of swine (Dwivedi et al., 2011). Pigs in group 3 received an IM injection of the commercial vaccine FluSure XP (Zoetis) at the label dose. Group 4 received empty nanodiscs (eND) formulated as Groups 1 and 2, respectively. All groups were booster vaccinated 18 days later by the same route and antigen/adjuvant combination as the initial immunization. Serum samples were collected before the first immunization and 10 days after the booster immunization. Peripheral blood mononuclear cells (PBMCs) and bronchoalveolar lavage fluids (BALF) were collected from IN immunized animals 10 days after booster immunization under anesthesia via endotracheal lavage as previously described (Hennig-Pauka et al., 2007).

### 2.4. Pig study 2

The second experiment was designed to ascertain the ability of M2ND to expand the breadth of protective immunity afforded by a monovalent IAV WIV vaccine using an H3 mismatched pair of vaccine and challenge IAV (Bolton et al., 2019). This was accomplished by vaccinating pigs with a monovalent IAV WIV vaccine prepared using the NY/11 virus, which expresses the antigenic motif NYNNYK that belongs to the H3 Red antigenic cluster and challenging them with the H3 mismatched MN/16 virus, which expresses the disparate antigenic motif KYNNYK that belongs to the H3 Green antigenic cluster (Bolton et al., 2018). Both viruses are derived from the same 1998 introduction/lineage and carry the same pM gene segment derived from the A/(H1N1) pdm09 virus; hence, they express the pM2 isoform. This study involved a total of 41 pigs. Pigs in group 1 (n=8) received a 2 ml IM injection of WIV vaccine with 250 HAU units of NY/11 virus adjuvanted with 20 % (v:v) of Emulsigen-D (MVP Laboratories) concurrently with a 2 ml IM injection at an adjacent site of 90 μg M2ND adjuvanted with ISA206 at 1:1 ratio. Pigs in group 2 A (n=8) and Group 2B (n=5) received a 2 ml IM injection of the same WIV vaccine prepared with the NY/11 virus as stated above. Pigs in group 3 (n=8) received a 2 ml IM injection with 90 μg M2ND adjuvanted with ISA 206 at 1:1 ratio. Pigs in group 4 (n=4) served as a mock-vaccinated group by receiving a 2 ml IM injection of saline mixed with Emulsigen-D at 20 % (v:v), and 2 ml IM injection at an adjacent site of empty NDs (eNDS) mixed with ISA206 at 1:1 ratio. Pigs in group 5 (n=4) also served as a mock-vaccinated group by receiving a 2 ml IM injection of vaccine diluent (saline) mixed with 20 % (v:v) of Emulsigen-D. At 21 days after the initial vaccination, all groups were given a booster vaccination using the same original route, volume, and antigen/adjuvant mixture. Pigs in group 6 (n=4) served as a strict control group that was neither vaccinated nor challenged. Sixteen days after the booster vaccination, groups 1, 2 A, 3, and 4 were challenged intratracheally (IT), under anesthesia, with 10^6^ PFU of the H3 antigenically mismatched MN/16 virus as previously described (Larsen et al., 2000; Vincent et al., 2009). As a control to assess the efficacy of the H3 antigen matched vaccine, pigs in groups 2B and 5 were challenged in the same manner with 10^6^ PFU of the NY/11 virus. After viral challenge, all pigs were monitored daily for clinical signs. Pigs were sedated and euthanized five days after the virus challenge via IV injection (Euthasol), and their lungs were removed. The percentage of the lungs affected with pneumonia (purple-red consolidation typical of IAV) was scored by a pathologist blinded to the treatments. The total percentage of the lungs affected with pneumonia was calculated based on weighted proportions of each lobe with respect to the total lung volume as previously described (Halbur et al., 1995). Bronchoalveolar lavage fluids (BALF) were collected as previously described (Larsen et al., 2000), and the virus load determined using a PFU assay and reported as PFU/ml as previously described (Baker, 2022).

### 2.5. Pig study 3

The third experiment was designed to test the ability of M2NDs delivered IM to work as a stand-alone vaccine capable of conferring protective immunity against an IAV challenge. Two groups of pigs (each n=7) were immunized IM twice at a 21-day interval with either M2ND displaying a total 90 μg of M2 per dose or with eNDs. Before administration, the M2ND and eNDs were mixed with Emulsigen-D at 20 % (v:v) in a 2 ml volume. A third group (n=2) was mock-vaccinated with vaccine diluent (saline) mixed with Emulsigen-D. A fourth group (n=2) was neither vaccinated nor challenged, serving as the strict control. Sixteen days after the booster vaccination, the pigs in all three groups were challenged intratracheally (IT) with 10^6^ PFU of MN/16 as described above. The pigs were monitored daily for clinical signs, and nasal swabs were collected before viral challenge and at 3- and 5-days post challenge. Five days after the challenge, the animals were euthanized, and their lungs removed. The percentage of the lungs affected with pneumonia was scored as described above. The extent of virus load on the nasal swabs and BALF was determined using the PFU assay as previously described (Baker, 2022) and are reported as PFU/ml.

### 2.6. Pig study 4

The fourth experiment was designed to assess the ability of M2ND delivered both IM and IN to confer protective immunity against IAV. To enable the delivery of the vaccine via these two routes using the same adjuvant, the type A CpG-ODN D19 (Kamstrup et al., 2001) was incorporated into the nanostructures. Three groups of pigs (n=9 per group) were immunized via both IN and IM routes twice at a 4-week interval with 2 ml volume per route of the following vaccine formulations: Group 1 pigs received M2ND-CpG-ODN; group 2 pigs received eND-CpG ODN; and group 3 pigs received eND-CpG-ODN mixed with M2 (eND-CpG-ODN + M2). The NDs in all three formulations were adjuvanted identically by incorporating 10 μg of CpG ODN D19 per vaccine dose. The combined total dose of M2 delivered to each pig IN and IM per immunization was 50 μg (25 μg/route). Each group was boosted with the same respective vaccine formulation 28 days after the initial vaccination. Seventeen days after the booster, the animals in all three groups were challenged IN with 10^6^ PFUs of MN/16, using an Ideal Prima Vaccinator fitted with an Ideal^®^ Prima^®^ Mist Sprayer (Neogen, Lansing, MI), and monitored for clinical signs for five days. For this experiment, the route used to perform the virus challenge was changed from the IT route used in the previous experiments to the more natural and simpler IN route. This change was done based on results of a study performed before this trial showing that challenging pigs via the IN route with 10^6^ PFU of MN/16 was as efficient in infecting both the upper and lower respiratory tracts as the same dose delivered IT (data not shown). Nasal swabs were collected before virus challenge and at 3- and 5-days post challenge. PBMC and serum were collected before challenge, and at 5 days post challenge. All pigs were euthanized and necropsied at 5 days after the challenge, and their lungs were harvested. The percentage of lung affected with pneumonia was scored as described above. BALF fluids were collected as previously described (Larsen et al., 2000), and the extent of virus load in BALF and nasal swabs was determined as previously described (Baker, 2022) and are reported as PFU/ml.

### 2.7. M2 and M2 ectodomain (M2e)

A consensus sequence of the M2 protein was generated using FASTA software based on the analysis of 3816 sequences of IAV isolated from swine in North American in 2019, which are available in the Influenza Research Database (Zhang et al., 2017). The resulting consensus M2 sequence used in this study is: MSLLTEVETPTRSEWECRCSDSSDPLAIAANIIGILHLILWITDRLFFKCIYRRFKYGL

KRGPSTEGVPESMREEYQQEQQSAVDVDDGHFVNIELE. Except for the amino acid substitution of V27A, the consensus sequence is identical to the sequence of the pM2 isoform expressed by the A/(H1N1) pdm09 virus, which caused the human influenza pandemic in 2009 (Garten et al., 2009), and is the predominant isoform of M2 expressed by IAV currently circulating in swine (Arendsee et al., 2021). A synthetic peptide with amino acid sequence of the M2 ectodomain (M2e) of the pdm M2 (pM2) isoform (MSLLTEVETPTRSEWECRCSDSSD) was obtained from GeneScript. For all four studies, the M2NDs were prepared using the pM2 isoform, which is the same isoform expressed by the two H3N2 strains (NY/11 and MN/16) used to challenge the animals.

### 2.8. Construction of M2 expression plasmid and protein expression

A vector containing the consensus M2 sequence listed above was constructed by cloning the synthetic gene into pET28a vector between Nco1 and Hind III sites (NEB). The gene was ordered from IDT DNA, the DNA sequence was optimized for E. coli expression. To simplify the purification and detection of the M2 protein, a N-terminal polyhistidine tag followed by TEV protease recognition site was added to the sequence. After cloning, the DNA sequence was confirmed by sequencing performed by ACGT Inc. Competent cells BL-21gold (DE3) were obtained from Agilent (Santa Clara, CA) and used for protein expression. For expression, 400 ml of TB medium with kanamycin (30 mg/L) in 2 L Fernbach flasks was inoculated with 5 ml of the overnight starting culture and incubated at 37 °C at 220 RPM until an OD600 of 1.0 was reached. At that point, the bacterial culture was induced with 0.5 mM IPTG. After 18 h of incubation in a 25 °C incubator, the cells were collected by centrifugation and stored at −80°C until further processing.

### 2.9. M2 Purification

The cell pellet from 4.8 L culture was resuspended in 400 ml PBS supplemented with 3 % Empigen BB, sonicated, and solubilized for 1 hour at 4 °C. The lysate was clarified by centrifugation at 35,000 RPM (Ti-45 rotor). Solubilized material was mixed with 5 ml Ni-NTA resin, incubated 1 hour on ice, then the resin was transferred to the chromatography column, washed with buffer containing 40 mM imidazole and 0.3 % Empigen, and eluted with 0.5 M imidazole. The sample was concentrated by ultrafiltration and the buffer was exchanged to 40 mM Tris/HCl pH 7.4, containing 0.1 % empigen BB and 1 mM DTT. The protein was loaded on an ion-exchange column (Protein-Pak DEAE 15 HR 10×100 mM), equilibrated with the same buffer, and purified in NaCl gradient (0–0.4 M) in 5 column bed volumes. The fractions were analyzed by SDS-PAGE and those with an estimated M2 purity exceeding 85 % were combined and used to prepare the vaccine.

### 2.10. Preparation of nanodiscs incorporating M2 (M2ND)

The incorporation of M2 into the lipid bilayer of the nanodiscs was accomplished following our previously described standard protocols to assemble nanodiscs (Denisov and Sligar, 2016; Luthra et al., 2013; Ritchie et al., 2009). Briefly, the assembly process involves detergent solubilizing the membrane scaffold proteins (MSP) together with the desired synthetic phospholipid (PL) or a mixture of lipid types. This study used either the saturated lipid DMPC or the lipid with a single unsaturated side chain DOPC. The M2 samples were mixed with a nanodisc reconstitution mixture (MSP1D1(−):DMPC:cholate molar ratios 1:75:150) prepared as previously described (Luthra et al., 2013). For every mg of M2, approximately 8 mg of MSP were used. After a short incubation, the detergent was removed with amberlite XAD-2 resin. Then the samples were filtered, loaded onto Ni-NTA column, washed with buffer containing 20 mM imidazole and eluted with buffer containing 0.5 M imidazole. The samples were analyzed using size-exclusion chromatography followed by SDS-PAGE analysis.

### 2.11. Quantitation of M2 incorporated into ND

The concentration of M2 incorporated in NDs was determined by image analysis of SDS-PAGE gels using BSA as a standard. For analysis, 6 gel lanes of the gel were used for calibration; one for molecular weight standard; and the rest for the M2 and/or M2 nanodisc samples. Typically, 100, 200, 400, 750, 1000 and 2000 μg of BSA were loaded and 6–8 repeats of the M2 sample were resolved using a 4–20 % precast Mini-Protean TGX gels (Bio-Rad, Hercules, CA). The gels were stained with AcquaStain (BulldogBio, Portsmouth, NH) for 1 hour and destained with distilled water overnight. The imaging was performed with IBright imager (Thermofisher Scientific). The analysis to determine the concentration of M2 was done with either ImageJ or GelAnalyzer software (www.gelanalyzer.com).

### 2.12. Preparation of M2NDs and eNDs with CpG-ODN

The type A CpG-ODN D19 (Kamstrup et al., 2001) with a 3′ Cholesterol-modified DNA oligo (5’-ggTGCATCGATGCAGgggg/3CholTEG/−3’) was ordered from Integrated DNA Technologies (Coralville, Iowa). Before mixing adjuvant with either eNDs or M2NDs samples, 100 μM DNA solution in sterile water was heated at 50 °C for 10 minutes. The DNA concentration was confirmed by UV spectra. To prepare the samples for administration, the CpG-ODN adjuvant solution was combined with either M2NDs or eNDs, incubated at 37 °C for 15 minutes to facilitate incorporation of the cholesterol-modified DNA into the lipid bilayer, and diluted with sterile PBS to the final concentration. The incorporation of the CpG-ODN into M2ND or eNDs was confirmed by size-exclusion chromatography.

### 2.13. Immunostimulatory activity of M2ND-CpG-ODN assemblies

The bioactivity of CpG-ODN D19 (CpG-ODN) incorporated into the M2ND assemblies (M2ND-CpG-ODN) was determined by assessing the ability of these structures to stimulate the production of IFN-α by freshly isolated porcine peripheral blood mononuclear cells (PBMC) as previously described (Calzada-Nova et al., 2011; Domeika et al., 2004). Briefly, 5–7 ml of venous blood was collected in BD vacutainer heparin tubes, and PBMC isolated by isopycnic centrifugation using Histopaque 1077 (Sigma-Aldrich). PBMC were cultured in a 96-well plate at 5×10^5^ per well in a 200 ml volume and left untreated or exposed to either M2ND-CpG-ODN or CpG-ODN D19 at a 2.5 mg/ml dose. Exposure to pseudorabies virus (PRV) at a MOI=0.05 was used as a positive control. After 18 h of culture, the amount of IFN-α present in the supernatants was determined using a porcine IFN-α specific ELISA as described previously (Calzada-Nova et al., 2010). The presence of IFN-α in serum was detected using the same IFN-α specific ELISA as above.

### 2.14. ELISA

The presence of IAV-, M2- and M2e-specific antibodies in serum and bronchoalveolar lavage fluids (BALF) were determined by ELISA as previously described (Larsen et al., 2000), with some modifications. The IAV antigen consisted of purified and UV inactivated A/swine/Illinois/A02090306/2015 (H3N2) which belongs to the H3 clade IV-A and has the pdm M2 gene. Virus diluted to a HA concentration of 100 HA units/50 μl was used to coat Immulon-2 96-well plates (Dynex, Chantilly, VA) and incubated overnight at 4°C. Plates were blocked with 100 μl of 10 % BSA in PBS and washed three times in PBS with 0.05 % Tween 20 (PBS-T) (Sigma). Alternatively, either recombinant M2 protein produced in *E. coli*, or the synthetic M2e peptide, both described above, were used as the target antigens to coat the plates. Serum or BALF samples were added (50 μl aliquots) in triplicate at appropriate dilutions. Positive control samples and appropriate negative controls were run on each plate. Plates were incubated at room temperature for 1 h, washed with PBS-T and incubated with mouse anti-swine IgA monoclonal antibody (mAb, unconjugated; Serotec, Raleigh, NC) or peroxidase-labeled goat anti-swine IgG (Kirkegaard and Perry, Gaithersberg, MD). The IgA ELISAs were then developed by the addition of horseradish peroxidase conjugated rabbit anti-mouse IgG (Zymed, San Francisco, CA). After washing, specific antibody binding was revealed by the addition of TMB/peroxidase substrate (Kirkegaard and Perry). Antibody titers are defined as the reciprocal of the highest dilution of sample for which the optical density (OD) was at least two times the OD of the negative control serum or BALF samples run on the same plate.

### 2.15. Enzyme-linked immunospot (ELIspot) assay for porcine Interferon (IFN)-γ

The presence of the M2-, M2e, and IAV-specific IFN-γ-secreting cells (SC) in PBMC was determined using an IFN-γ ELIspot assay as previously described (Zuckermann et al., 1998; Zuckermann et al., 1999; Larsen et al., 2000; Meier et al., 2003). Briefly, peripheral blood mononuclear cells (PBMC) were obtained from swine at the indicated time after immunization or IAV virus challenge. Immulon^®^ 2 plates (96 well) were coated overnight at 4°C with mAb P2G10 specific for porcine IFN-γ (Mateu de Antonio et al., 1998) in carbonate buffer (pH 9.6). To each well, 5×10^5^ PBMC in 100 ml were added and mixed in triplicates with either 100 μl plain media (RPMI+5 % FBS 2 mM glutamine, l5 mM HEPES, 100 U/ml penicillin and 100 μg/ml streptomycin); media containing IAV (1:500 dilution of FluSure XP antigen); 5 μg of M2; or 10 μg of M2e peptide. The concentration of viral antigens used to stimulate the cells were those found to optimally stimulate an IFN-γ-SC response of PBMC from IAV and M2ND immunized animals. As a positive control of the assay, wells containing 5×10^4^ PBMC in 100 ml were stimulated with 100 ml of 1 μg/ml phytohemagglutinin (PHA). The plates were then incubated for 18 h at 37°C in 5 % CO_2_, washed with PBS-T, incubated with a second biotin-conjugated mAb P2F6 specific for porcine IFN-γ (Mateu de Antonio et al., 1998) for 2 h at room temperature, washed again and incubated with HRP-conjugated streptavidin (Kirkegaard and Perry) for 1 h at room temperature. Following a final wash, the plates were incubated with TMB membrane substrate (Kirkegaard and Perry) for 10 min at room temperature and washed once with distilled water. The average numbers of spots counted in wells cultured with media alone were subtracted from the average number of spots counted in wells stimulated with viral antigen. The numbers of IFN-γ-SC cells are reported as number of spots/5×10^5^ PBMC.

### 2.16. Statistical analysis

Statistical analyses were done using Student’s t-test or Kruskal-Wallis One-Way ANOVA with GraphPad Prism 9 software.

## 3. Results

### 3.1. Assembly of M2NDs

**Fig. 1.**
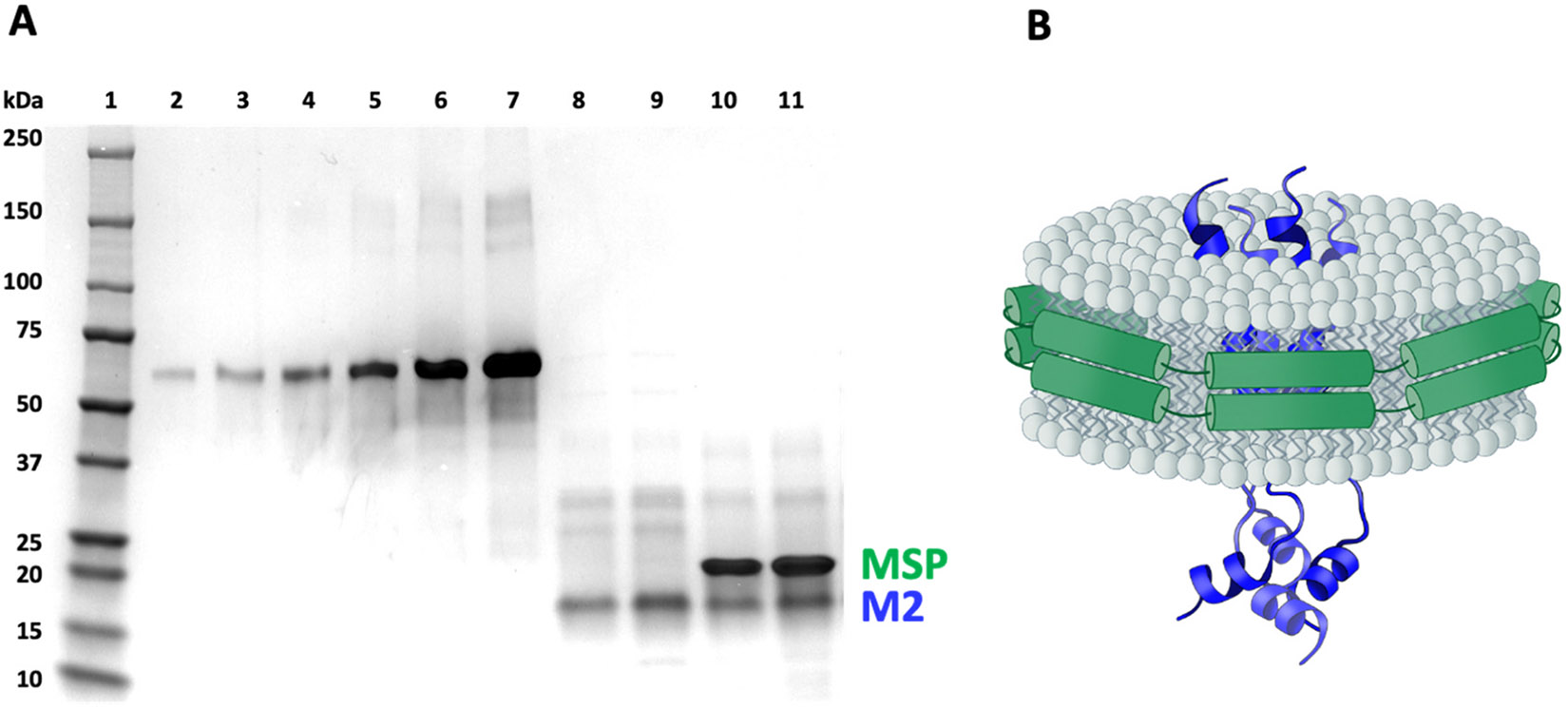
M2ND assemblies separated into their discrete-sized protein components by SDS-PAGE. (A) Purified M2, M2ND, and bovine serum albumin (BSA) standards were resolved by SDS-PAGE and visualized using AcquaStain. Lane 1- molecular weight protein markers (Precision Plus, BioRad); lanes 2–7 - BSA at 62, 125, 250, 500, 1000, 2000 μg per lane, respectively; lanes 8 and 9 - purified M2 samples; lanes 10 and 11 - M2NDs. The amount of M2 embedded in the ND assemblies was determined using the density of the M2 band as compared to the density of the BSA standards as described in Materials and Methods. (B) Model of a ND particle that contains a phospholipid bilayer encircled by two membrane scaffold proteins (MSP) with an embedded tetrameric M2.

Incorporation of purified M2 into NDs (M2NDs) was achieved by mixing M2 with ND reconstitution mixture at the molar ratios described in materials and methods (Fig. 1). Initial batches of M2NDs were assembled using a large excess of the reconstitution mixture, which allowed us to use the same lipid to MSP ratio used to assemble “empty” ND (eND) devoid of M2 protein. After short incubation, the detergent was removed with amberlite XAD-2 resin. Then the samples were filtered, loaded onto Ni-NTA column, washed with buffer containing 20 mM imidazole (without detergent), and eluted with buffer containing 0.5 M imidazole. Excess of MSP and NDs not containing M2 (eNDs) were removed during the washing step. The samples were separated using size-exclusion chromatography (not shown) followed by SDS-PAGE to analyze the resolved fractions. Fractions showing the highest amounts of M2 were pooled and used as the M2ND immunogen. SDS-PAGE analysis of the combined chromatography fractions corresponding to size 10.5–14 nm, contained both M2 and MSP indicating that M2 was successfully incorporated into NDs (Fig. 1). The concentration of M2 incorporated in NDs was determined by image analysis of SDS-PAGE gels using BSA as a standard (Fig. 1). The M2 protein in the assembly was stable in the absence of detergent for a prolonged period and could be diluted, concentrated, subjected to several freeze-thaw cycles without loss of the material. This contrasts with M2 which deteriorates within a day in the absence of detergent (data not shown).

### 3.2. Pig study 1: Immunogenicity of M2NDs

**Fig. 2.**
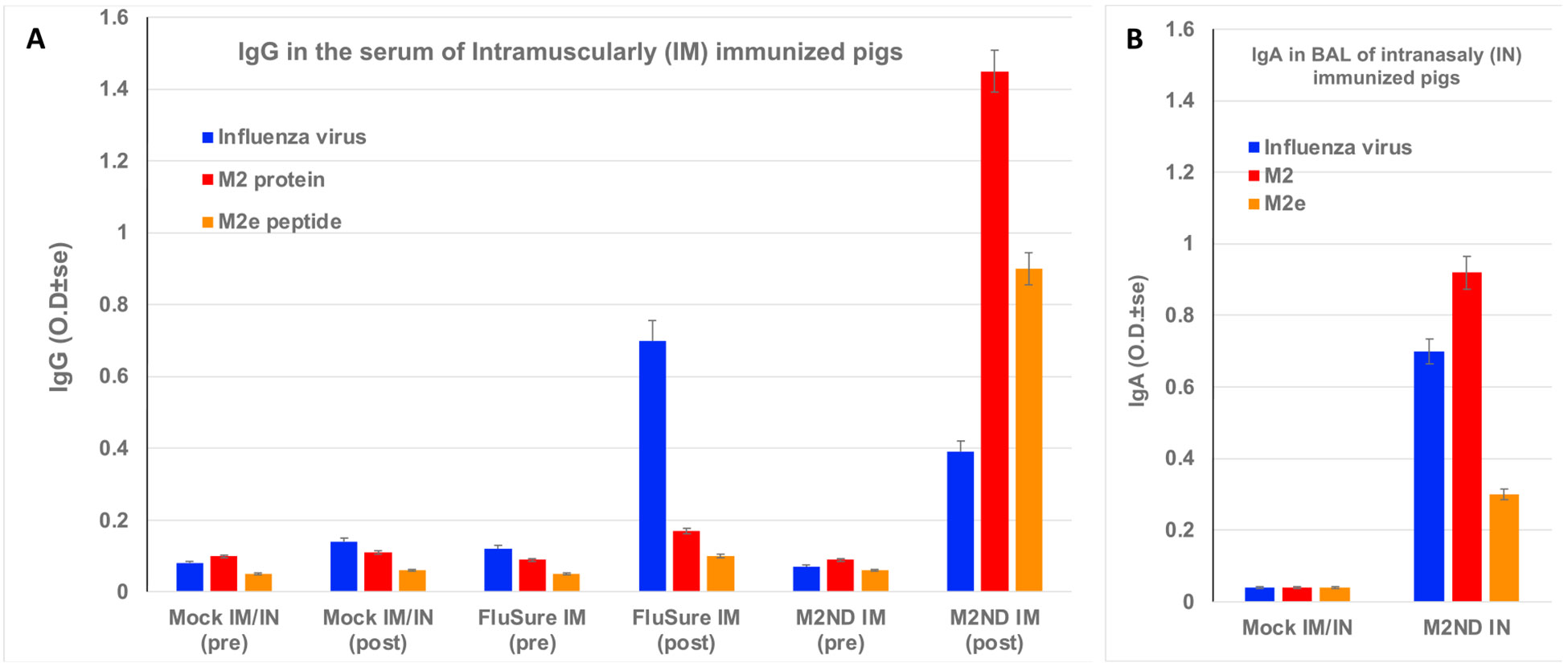
Serum IgG and mucosal IgA responses to M2 elicited by M2NDs (Pig study 1). Two groups of four-week-old pigs (n=4) were immunized with M2NDs either intramuscularly (IM) or intranasally (IN) twice at a two-week interval. Following the same immunization schedule, a third group was vaccinated IM with FluSure XP. A fourth group was mock vaccinated, both IN and IM with empty NDs not displaying M2 (eNDs). (A) IgG levels in the serum of pigs before (pre) and after (post) booster IM immunization with either M2NDs, eNDs or FluSure XP. (B) IgA levels on the BALF of pigs 10 days after a IN booster immunization with M2NDs or eNDs. Immunolon II 96 well-plates were coated with either UV inactivated influenza virus, synthetic M2e peptide (M2e), or recombinant M2 protein. Serum or BAL samples in triplicate were assayed using an ELISA for the presence of IgG or IgA binding to the indicated antigen using HRP-labeled rabbit anti porcine IgG or IgA antibodies. The amount of bound reagent was revealed by the addition of TMB substrate. Data represents the average O.D. ± SE minus the background of samples from three to four pigs per treatment.

**Fig. 3.**
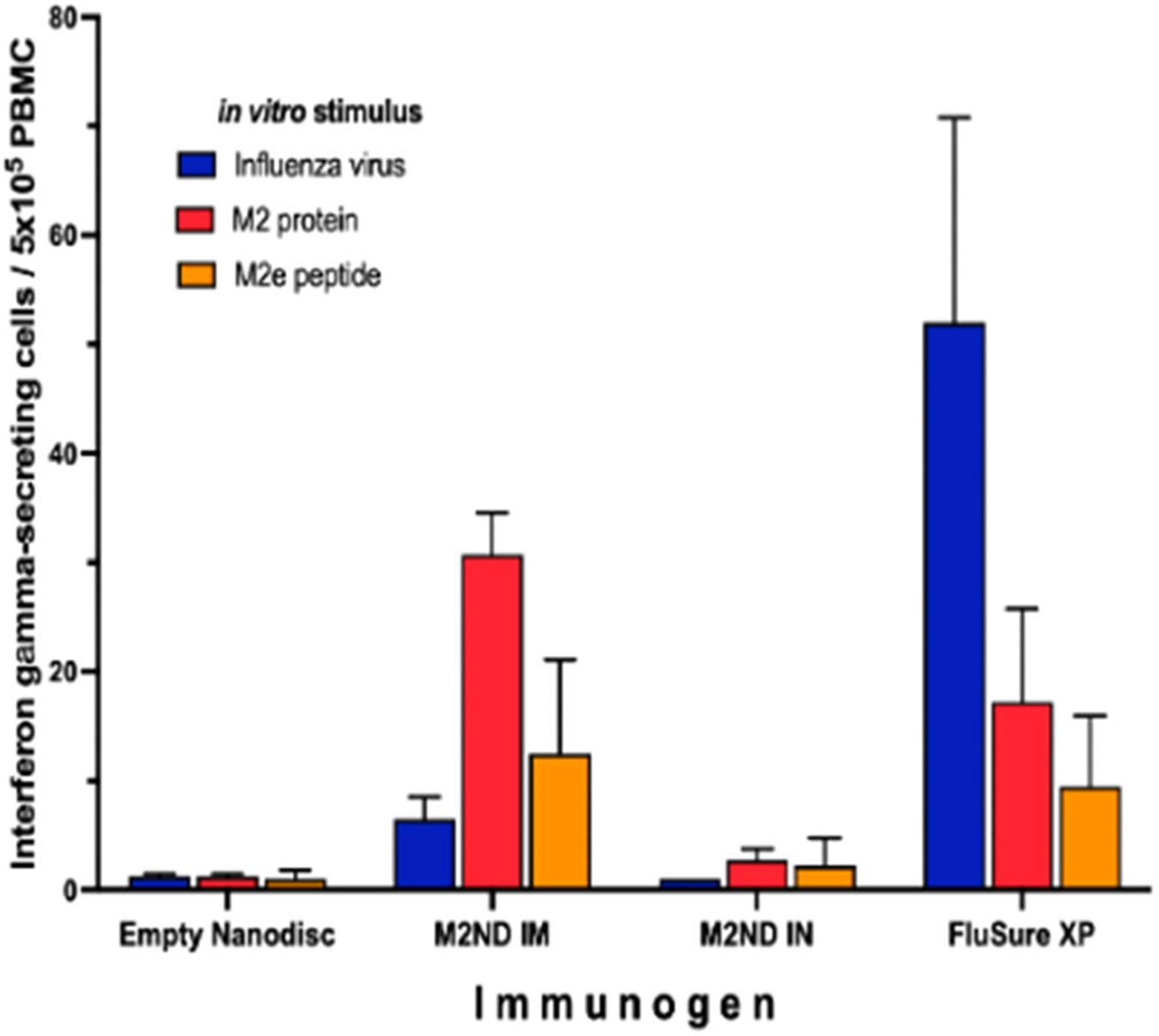
Cell-mediated immune response to M2 elicited by M2NDs (Pig study 1). Peripheral blood mononuclear cells (PBMC) were isolated from the same groups of pigs listed in Fig. 2 ten days after the second immunization. PBMC were stimulated in triplicate for 8 h with either FluSure XP, recombinant M2, or synthetic M2e peptide (M2e). The number of cells producing interferon-γ in response to these stimuli were enumerated using an Interferon-γ-ELISPOT. Data represents the average ± SE response of four pigs per treatment minus the background spontaneous response.

The immunogenicity of M2 displayed in NDs was examined by immunizing swine twice at an 18-day interval, either intramuscularly (IM) or intranasally (IN), with M2ND containing 45 μg of M2. As a positive control, a group of animals was IM vaccinated with a commercial IAV inactivated whole virus vaccine (FluSure XP). None of the animals in the trial exhibited the presence of IgG in their serum capable of reacting with either M2, M2e, or influenza virions before vaccination, as detected by ELISA. (Fig. 2A, samples labeled as pre). At 10 days after the IM booster immunization with M2ND (group 1), the serum of these animals exhibited the presence of IgG reacting with M2, M2e, as well as with influenza virions (Fig. 2A, samples labeled as post). The BAL collected from animals 10 days after the IN-booster immunization with M2NDs (group 2) exhibited the presence of IgA that reacted with recombinant M2, M2e, as well as influenza A virions (Fig. 2B). The serum of the animals immunized with FluSure XP (group 3) exhibited the presence of IgG reacting with influenza virions but not with M2 or M2e. The serum and BALF obtained from the mock-immunized pigs (group 4), which were immunized IM and IN with eND, did not exhibit the presence of serum IgG (Fig. 2A) or IgA in their BALF (Fig. 2B) specific for any of three antigens tested. PBMC isolated from animals ten days after the booster IM immunization M2NDs (group 1), contained interferon-γ secreting cells (IFN-γ-SC) responsive to stimulation with M2 (Fig. 3). IFN-γ-SC responsive to influenza virus or to the M2e peptide were also detectable, but at a lower frequency. PBMC isolated from animals immunized IN with M2ND (group 2) did not exhibit a detectable IFN-γ-SC response in the blood. PBMC isolated from animals vaccinated with the FluSure XP (group 3) exhibited a marked IFN-γ-SC response to influenza virus and a relatively lower response to M2 or M2e. PBMC isolated from animals immunized IM with eND (group 4) did not exhibit a detectable IFN-γ-SC response to their stimulation with any of the three antigens tested (Fig. 3). Combined, these results indicate that IM immunization with M2ND stimulated the production of M2-specific IgG antibodies in serum capable of recognizing IAV virions and M2-specific IFN-γ-SC. On the other hand, IN immunization with M2NDs elicited M2-specific IgA response in the lower respiratory tract capable of reacting with influenza virus.

### 3.3. Pig study 2: M2NDs as a supplement to a conventional influenza WIV vaccine

Cell-mediated immunity (CMI) contributes to accelerated virus clearance and restricts disease progression (Sant et al., 2018). Since IM immunization of swine with M2NDs elicited a CMI response against M2, we examined the ability of M2NDs to expand the breadth of protective immunity afforded by a WIV vaccine. This notion was tested by taking advantage of the knowledge that a few amino acid differences in H3 antigenic clusters can affect the efficacy of WIV vaccines. Namely, vaccination of swine with a WIV vaccine prepared with an H3N2 virus is only capable of providing partial protective immunity from a challenge with a H3N2 virus expressing a disparate H3 antigenic cluster (Abente et al., 2018; Abente et al., 2019). Hence, we crafted experimental conditions that would result in the development of partial protective immunity induced by a WIV vaccine. As described in the materials and methods section, this was accomplished by vaccinating pigs with a monovalent IAV WIV vaccine prepared using the NY/11 virus, that belongs to the H3 Red antigenic cluster, and challenging them with the H3 mismatched MN/16 virus, that belongs to the H3 Green antigenic cluster (Bolton et al., 2018). Both viruses express the pM2 isoform. In the scenario of H3 antigenic mismatch, supplementation of the monovalent WIV influenza vaccine via a separate but concurrent immunization with M2NDs would test the ability of the immunity elicited by the M2ND vaccine to increase the anticipated partial protective immunity against a challenge with a IAV expressing an antigenically distinct H3 molecule. As a control for the efficacy of the vaccine under H3 antigen matched conditions, a group of animals was vaccinated with the WIV vaccine prepared with the NY/11 H3N2 virus and challenged with the same virus. In this scenario, in which the HA of the vaccine and the challenge virus are antigenically matched, a high level of protective immunity would be anticipated. Since in this scenario little room, if any, for vaccine efficacy improvement would be expected, the concurrent immunization with the M2NDs was not tested.

**Fig. 4.**
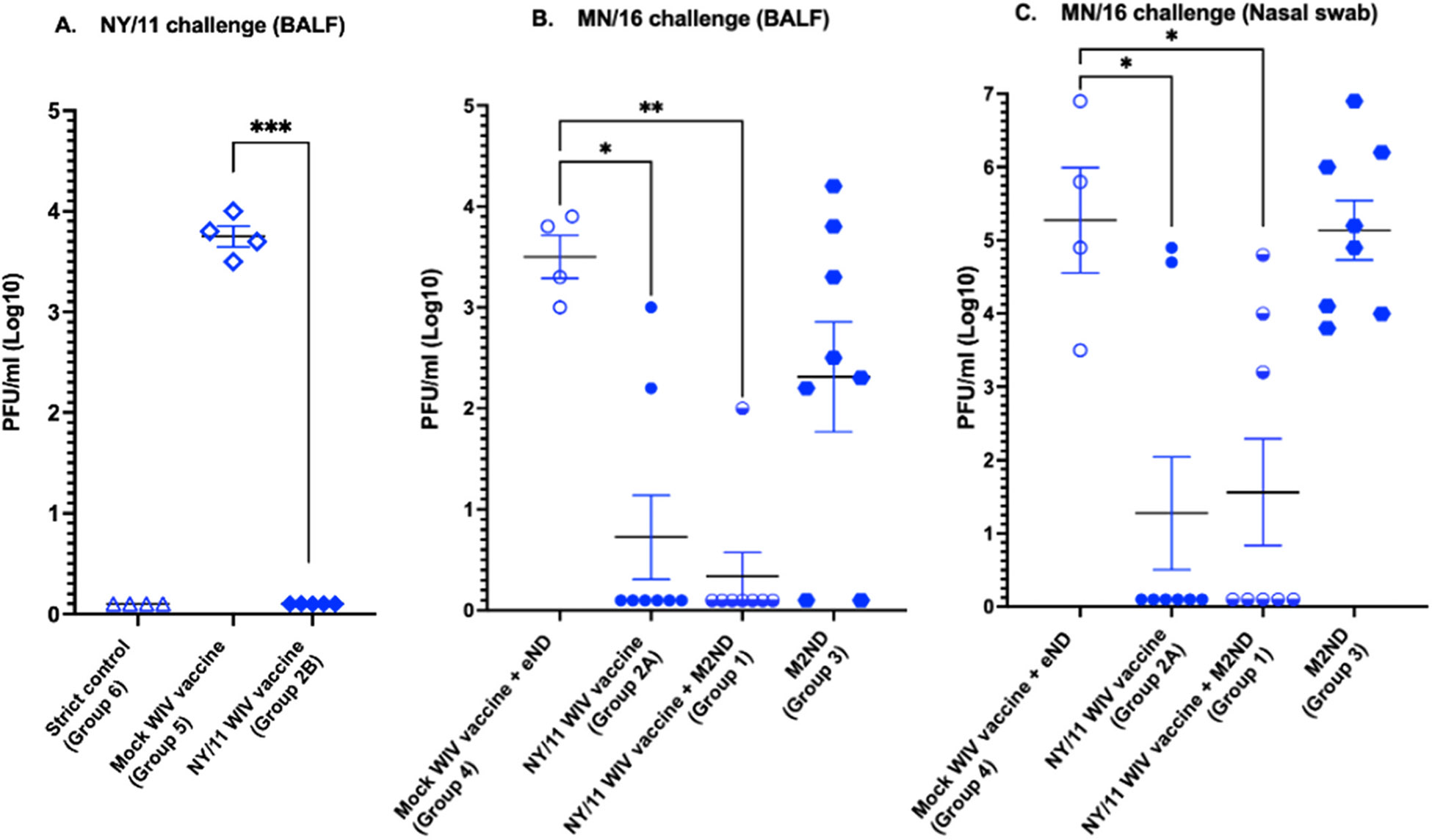
Viral load in the BALF and nasal secretions of H3N2 IAV challenged pigs previously immunized with a WIV IAV vaccine with and without a concurrent immunization with M2NDs (Table 1, Pig study 2). (A) *H3 antigen matched combination*. A pig cohort (Group 2B, n=5) was immunized IM twice at a 21-day interval with a whole inactivated virus (WIV) vaccine prepared using the NY/11 virus. A control group (Group 5, n=4) was mock vaccinated with vaccine diluent. Sixteen days after the booster immunization, pigs in groups 2B and 5 were challenged IT with 10^6^ PFU of NY/11. A strict control group (Group 6, n=4) was neither vaccinated nor challenged. (B and C) *H3 mismatched combination*. Groups of pigs were IM vaccinated twice at a 21-day interval with a WIV vaccine prepared with the NY/11 virus, with (Group 1, n=8) and without (Group 2 A, n=8) the concurrent IM injection of M2ND. An additional group (Group 3, n=8) was immunized only with M2NDs. A negative control group (Group 4, n=4) of pigs was mock-vaccinated with two separate injections consisting of vaccine vehicle (saline) mixed with the same adjuvant (Emulsigen-D) used for the WIV vaccine; and a second injection of empty ND without the M2 protein (eND) mixed with the same adjuvant used for M2ND (ISA 206). Sixteen days after the booster immunization, pigs in groups 1, 2 A, 3 and 4 were challenged IT with 10^6^ PFU of MN/16 virus. All the pigs in this trial were euthanized 5 days after the virus challenge and their lungs harvested. (A and B) Bronchoalveolar lung lavage fluids (BALF) and (C) nasal swabs were collected from the indicated groups 5 days after challenge and the viral load determined using a PFU assay. Each symbol represents the viral load of a single pig. The horizontal bars represent the mean ± SE of the group. Data were analyzed using (A) t-test and (B and C) Kruskal-Wallis test. Asterisks denote significant differences with the mock vaccinated group of their respective group: * p < 0.05, **p < 0.01, *** p <0.001. ns: no significant difference.

**Fig. 5.**
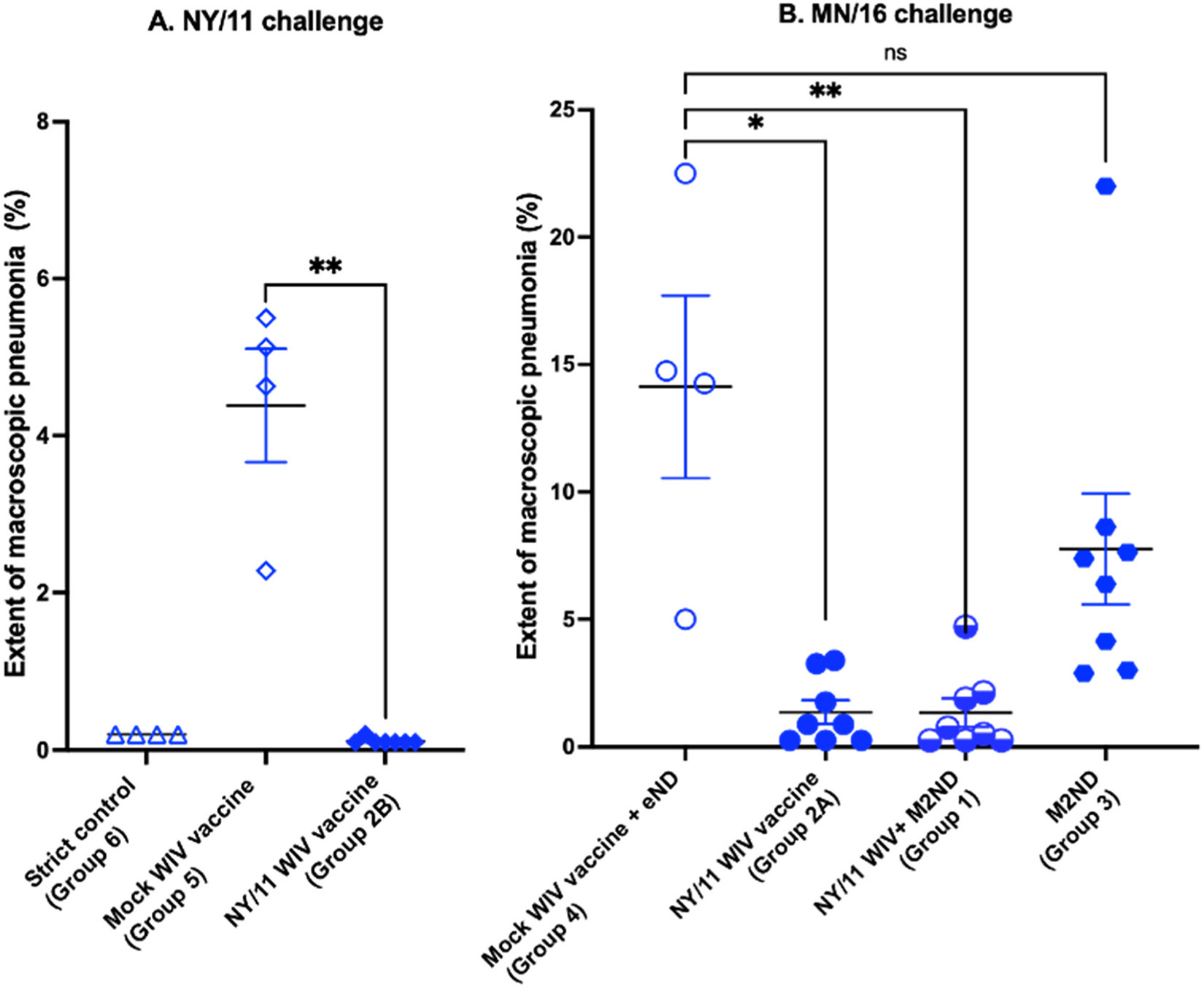
Extent of pneumonia in H3N2 IAV challenged pigs previously immunized with a WIV IAV vaccine with and without a concurrent immunization with M2NDs (Table 1, Pig study 2). (A and B) The same groups of pigs listed in Fig. 4 were euthanized 5 days after the virus challenge, their lungs removed and evaluated for the presence of pathological changes. The percentage of the surface area affected by pneumonia was visually estimated for each lung lobe. The total affected percentage of the entire lung was calculated based on the weighted proportions of each lob to the total lung volume. Each symbol represents the extent of pneumonia for each pig. The horizontal bars represent the mean ± SE of the group. The gross pathology scores were analyzed using the Kruskal-Wallis test. Asterisks denote significant differences between the mock-vaccinated and vaccinated groups or their respective group. * p<0.05, ** p<0.01. ns: no significant difference.

Except for a transient nasal discharge, challenge with either NY/11 or MN/16, even in mock-vaccinated pigs, did not induce any other clinical disease, such as sneezing, cough, lethargy, or loss of appetite (data not shown). Five days after virus challenge, the BALF collected from pigs in groups 4 and 5, which were mock-vaccinated and challenged respectively with either the NY/11 (Fig. 4A) or MN/16 (Fig. 4B) viruses, exhibited the abundant presence of infectious virus. The BALF collected from the lungs of pigs immunized with the NY/11 WIV vaccine and challenged with the same virus (Group 2B) had no detectable infectious virus (Fig. 4A), indicating complete protection (p<0.001). In addition, while the lungs of all the mock-vaccinated pigs challenged with NY/11 exhibited lesions typical of IAV-induced pneumonia, all but one of the pigs vaccinated with NY/11 WIV exhibited the complete absence of pneumonic lesions, and the one exhibiting lesions only had <0.5 % of the lung affected (Fig. 5A). The BALF (Fig. 4B) and nasal swabs (Fig. 4C) collected from 6 of the 8 pigs (75 %) immunized with the NY/11 WIV vaccine and challenged with the H3 mismatched MN/16 virus (Group 2 A) had no detectable infectious virus, indicating significant (p<0.05) but partial protection. The BALF collected from pigs in the group immunized with the WIV vaccine supplemented with M2ND (Group 1) exhibited increased protection (<0.01), as indicated by a higher number of pigs exhibiting the elimination of infectious virus (7 out of 8, 87.5 %) or a reduced viral load (Fig. 4B). However, an enhanced reduction in viral load in nasal swabs collected 5 days after virus challenge in Group 1 was not observed (Fig. 4C). Notably, as compared to the mock vaccinated pigs (Group 4), 2 of the 8 pigs (25 %) immunized only with M2NDs and challenged with MN/16 (Group 3) exhibited the complete elimination of infectious virus from their BALF, and three pigs had a noticeable reduction of their viral load (Fig. 4B). However, due to the variation observed in this parameter in the group vaccinated with M2ND alone, a statistically significant difference was not reached. As compared to the mock-vaccinated group challenged with MN/16 (Group 4), immunization with M2ND alone had no apparent effect in the viral load present in nasal secretions (Fig. 4C). Challenge of mock-vaccinated pigs with MN/16 (Group 4) resulted in a considerable amount of pneumonia, which was either eliminated or majorly diminished by immunization with the NY/11 WIV (Group 2 A), and supplementation of this vaccine with M2ND (Group 1) only improved slightly this parameter (Fig. 5B). Furthermore, immunization with M2ND alone (Group 3) did not significantly diminish the extent of pneumonia. Hence, the partial heterologous protection afforded by WIV vaccine in the H3 mismatched condition was modestly improved by supplementation with the M2ND vaccine, as evidenced by enhanced viral clearance in the lower but not upper respiratory tracts, and slight effect in preventing the emergence of pneumonia. The observation that immunization with M2ND alone eliminated or reduced the viral load from BALF in some animals provided enough impetus to further explore its potential ability to confer protective immunity.

### 3.4. Pig study 3: M2NDs as a stand-alone vaccine

To test the notion that the M2NDs could constitute a stand-alone IAV vaccine, we performed a follow-up vaccination and challenge experiment using M2NDs as the principal immunogen. To simplify the incorporation of the M2ND into the adjuvant and perhaps also increase the CMI response to M2, we changed the type of adjuvant used to formulate the M2NDs from the w/o/w ISA 206 used in the previous experiments to o/w Emulsigen-D, which is commonly used to formulate experimental vaccines against IAV for swine (Vincent et al., 2010; Abente et al., 2018). Animals in the study were vaccinated twice IM at a three-week interval with either M2ND (group 1) or, as a mock vaccine, empty NDs (eNDs) not displaying the M2 protein (group 2). Sixteen days after the booster vaccination, all animals in the trial were challenged with MN/16. As a control, a third group (group 3) was vaccinated with saline mixed with Emulsigen D and challenged with IAV at the same time as the other groups.

**Fig. 6.**
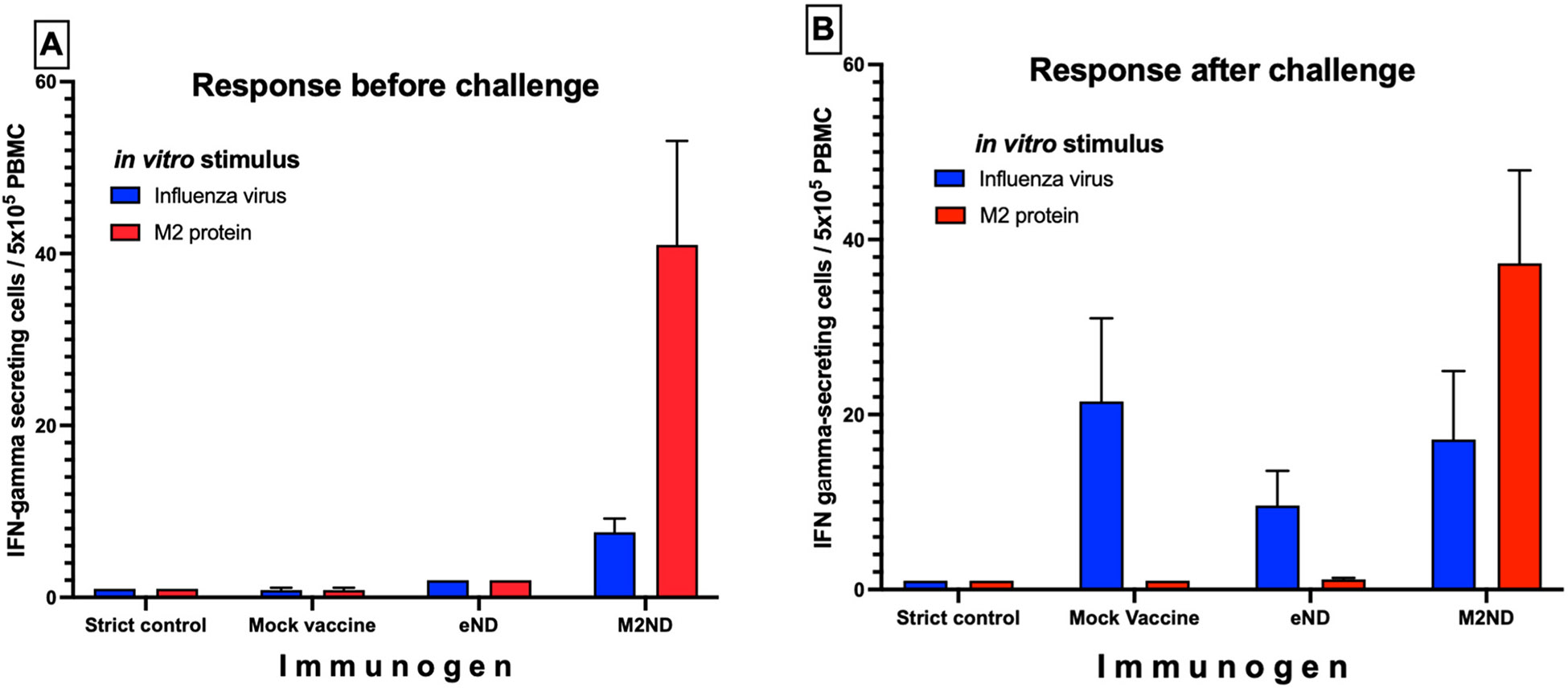
Frequency of M2- and IAV-specific IFN-γ-SC in PBMC from M2ND vaccinated swine before and after IAV challenge (Pig study 3). Two groups of pigs (n=7) were immunized IM with either M2NDs or eNDs twice at a two-week interval. A third group (n=2) was mock vaccinated with diluent, and a fourth group (n=2) was left untreated to serve as the strict control. (A) The day of IAV challenge (i.e., 16 days after the booster vaccination); and (B) 5 days after challenge, PBMC were isolated from these pigs. Triplicate cultures of the PBMC obtained from these pigs were stimulated for 8 h with either inactivated influenza virions or recombinant M2. The number of cells producing interferon (IFN)-γ in response to these stimuli were enumerated using an IFN-γ-ELISPOT. Data represent the average ±SE response of the PBMC from all the pigs in each treatment group minus the background spontaneous response.

**Fig. 7.**
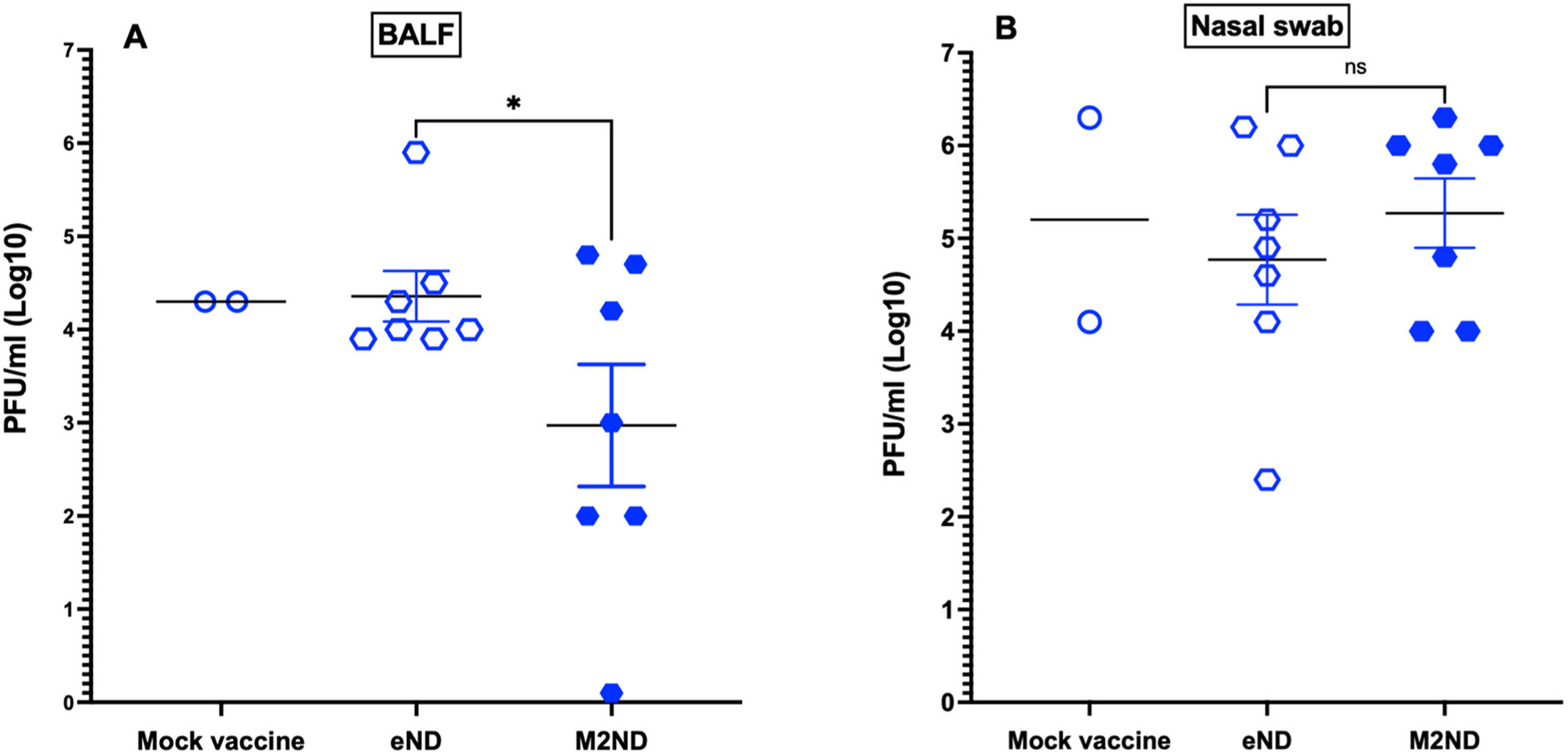
Viral load in nasal secretions and BALF from pigs vaccinated with M2ND and challenged with IAV (Pig study 3). The same groups of pigs listed in Fig. 6 were challenged 16 days after the booster vaccination with 10^6^ PFU of H3N2 MN/16. Nasal swabs were collected at 0 and 3 days after the challenge. Five days after the virus challenge, the animals were euthanized, their lungs harvested, and BALF collected. The titer of infectious virus in (A) BALF and (B) nasal swabs was determined using a PFU assay. Each symbol represents the viral load for each pig. The horizontal bars represent the mean ± SE of the group. Data were analyzed by using a t-test. Asterisks denote significant differences with the group vaccinated with eNDs: *p < 0.05. ns: no significant difference.

**Fig. 8.**
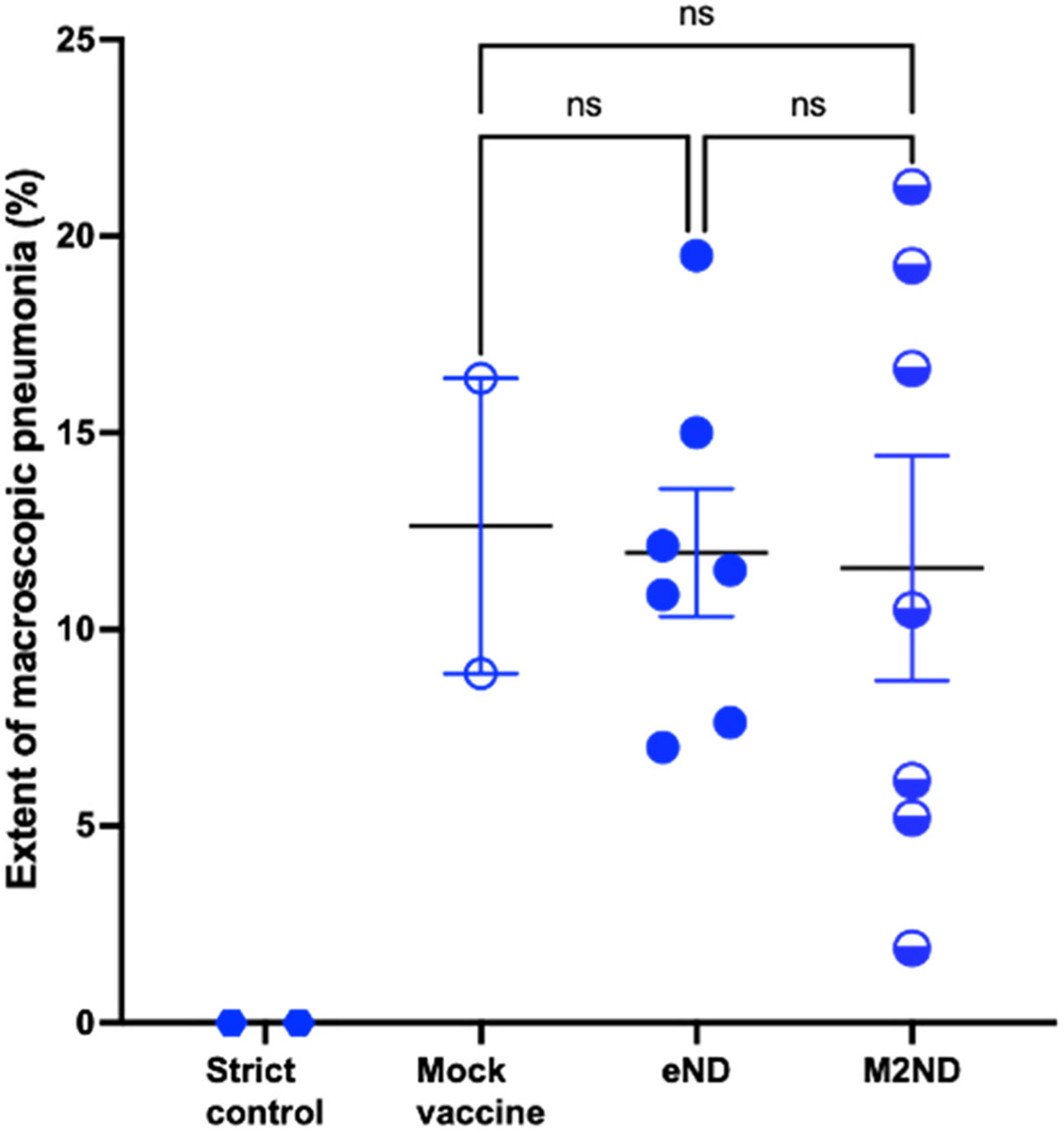
Extent of pneumonia in MN/16-challenged pigs previously vaccinated with M2NDs. (Pig study 3). The same groups of pigs listed in Fig. 6 were euthanized 5 days after the virus challenge, their lungs removed and evaluated for the presence of pathological changes. The percentage of the surface area affected by pneumonia was visually estimated for each lung lobe. The total affected percentage of the entire lung was calculated based on the weighted proportions of each lob to the total lung volume. Each symbol represents the extent of pneumonia for each pig. The horizontal bars represent the mean ± SE of the group. The gross pathology scores were analyzed using the Kruskal-Wallis test. Asterisks denote significant differences between the mock-vaccinated and vaccinated groups. ns: no significant difference.

Before the virus challenge, PBMC isolated from M2ND-vaccinated pigs stimulated with M2 exhibited the marked presence of IFN-γ-SC, and a lesser frequency in response to their stimulation with influenza virus (Fig. 6A). A similar frequency of IFN-γ-SC responding to M2 was observed in this group 5 days after the virus challenge and a slightly increased response to the influenza virus (Fig. 6B). Before virus challenge, PBMC isolated from animals in the groups that were vaccinated with either eND, saline, or left untreated (strict control) did not exhibit the presence of IFN-γ-SC in response to stimulation with M2. On the other hand, after virus challenge, pigs in the groups vaccinated with either eND or vaccine vehicle (saline) exhibited a detectable response to influenza virus (Fig. 6A and B). Except for a transient nasal discharge, challenge with either NY/11 or MN/16, even in mock-vaccinated pigs injected with vaccine diluent, did not induce any other clinical disease, such as sneezing, cough, lethargy, or loss of appetite (data not shown). The extent of protective immunity afforded by the M2ND vaccine was assessed by measuring the virus load in the BALF and nasal secretions present at 5 days after virus challenge. The BALF and nasal secretions of pigs in the group treated with eND exhibited a sizable viral load, which did not differ in magnitude from that exhibited by the pigs in the mock-vaccinated group (Fig. 7A and B). On the other hand, pigs in the group immunized with M2ND, exhibited a significant (p<0.05) decrease in the viral load present in their BALF compared to the group treated with eNDs (Fig. 7A). However, there was no difference in the viral load present in nasal secretions between any of the groups (Fig. 7B). As compared to the mock-vaccinated pigs, immunization with either eNDs or M2NDs did not affect the development of pneumonia resulting from the MN/16 challenge (Fig. 8).

### 3.5. Pig study 4: Efficacy of M2NDs adjuvanted with CpG-ODN administered IM and IN

**Fig. 9.**
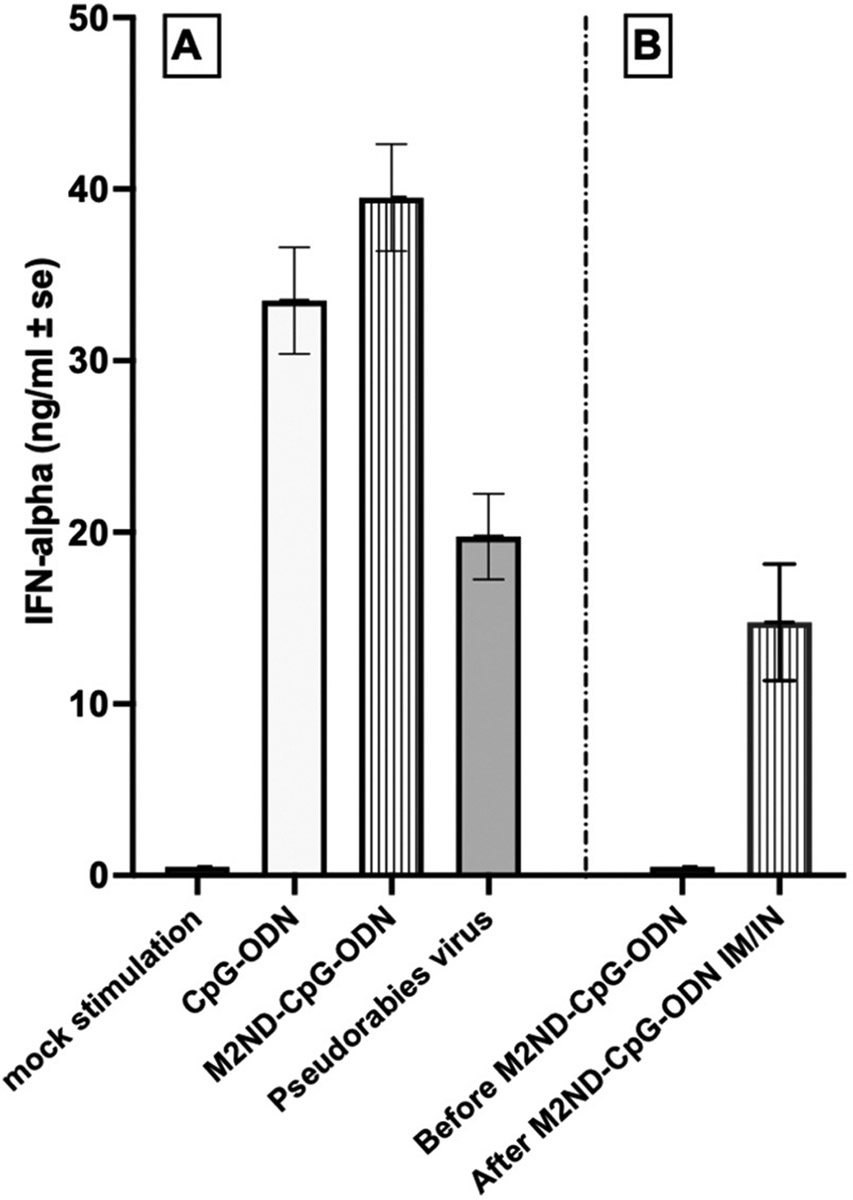
Interferon-alpha response to M2ND-CpG-ODN *in vivo* and *in vitro* (Pig study 4). (A) Porcine PBMC were cultured in the culture media (mock stimulation) or in the presence of 2.5 μg/ml of soluble CpG-ODN D19 or the equivalent amount bound to M2ND (M2ND-CpG-ODN). As a positive control, PBMC were also exposed to pseudorabies virus (MOI=0.05). After 18 h culture, cell free supernatants were harvested and examined for the presence of IFN-α. Data represent the mean ± SE of triplicate cultures. (B) A group of pigs was vaccinated via both IM and IN routes with M2ND-CpG-ODN. Serum samples were obtained from these animals immediately before and 2 days after being treated with the M2ND-CpG-ODN. Serum samples were assayed using a porcine IFN-α ELISA. Data represent the mean ± SE of IFN-α detected in the serum of four identically treated pigs.

The results presented above indicate that the IM immunization of swine with M2NDs prompts the generation of protective immunity in the lower but not in the upper respiratory tract and does not stop the development of pneumonia. Simultaneous IM and intranasal (IN) vaccination against IAV enhances protective immunity in pigs (Martini et al., 2021). Hence, to test the ability of M2NDs to stimulate protective immunity in both the upper and lower respiratory tracts, we performed an experiment in which the pigs were vaccinated both IM and IN with a modified formulation of the M2ND. Synthetic unmethylated CpG oligonucleotides (CpG-ODN) are potent Toll-like receptor-9 (TLR-9) agonists that have strong adjuvant properties (Kayraklioglu et al., 2021) and have been shown to increase the efficacy of an influenza vaccine administered IN (Tateishi et al., 2019). The ability of CpG-ODN to enhance the immunogenicity of NDs has been shown to work best when these immunostimulatory molecules are incorporated directly into the same NDs displaying the antigen (Fischer et al., 2013; Kuai et al., 2017). Hence, we modified the formulation of the M2NDs by incorporating directly into the nanostructures the type A CpG-ODN D19, which has been shown to induce the production of interferon-α (IFN-α), tumor-necrosis factor-α (TNF-α), and interleukin (IL)-12 by porcine PBMCs (Calzada-Nova et al., 2011; Kamstrup et al., 2001). Indeed, the incorporation of CpG-ODN D19 into the M2ND (M2ND-CpG-ODN) resulted in their ability to stimulate the production of IFN-α by porcine PBMC (Fig. 9A), which was comparable to that observed in response to their stimulation with an equivalent dose of free CpG-ODN (2.5 μg/ml). The ability of CpG-ODN incorporated into the M2ND structures to stimulate the production of IFN-α *in vivo* was evidenced by the presence of IFN-α in the serum of pigs two days after immunization with the M2ND-CpG-ODN vaccine (Fig. 9B).

**Fig. 10.**
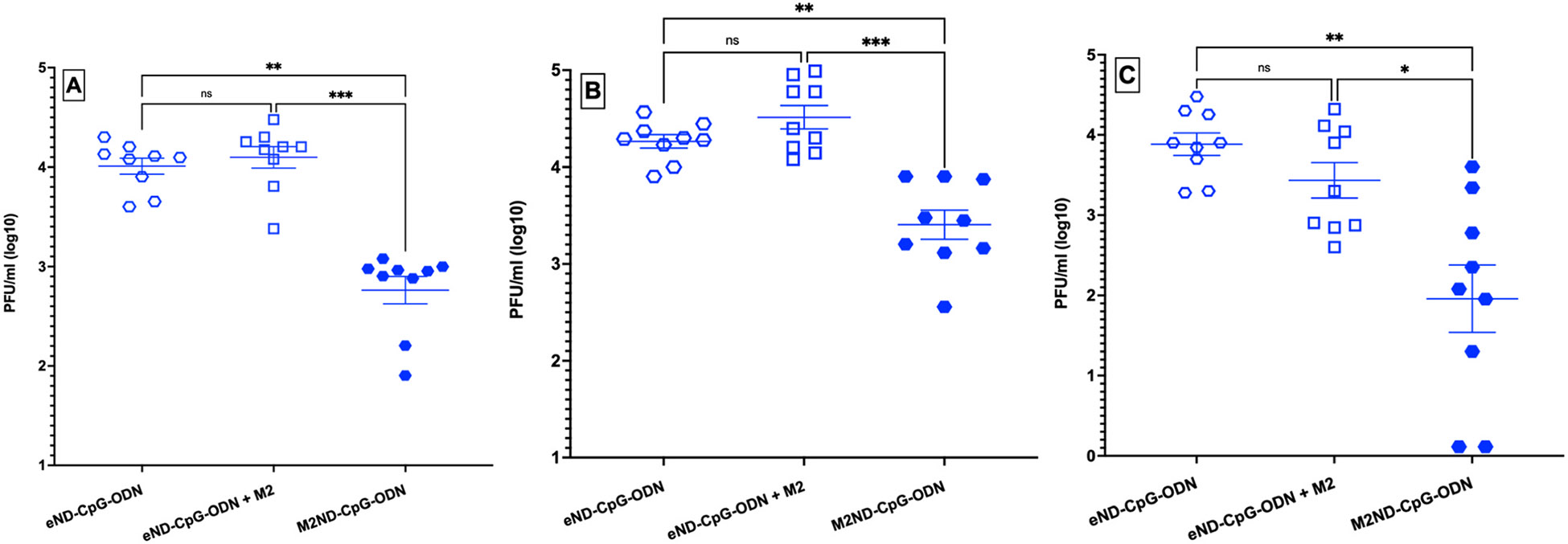
Viral load in nasal secretions and BALF from pigs vaccinated with M2ND-CpG-ODN and challenged with IAV (Pig study 4). Groups of pigs (n=9 each) were vaccinated IM and IN twice at a 3-week interval with either eND-CpG-ODN, eND-CpG-ODN + M2, or M2ND-CpG-ODN. The dose of M2 delivered by each route displayed in M2ND-CpG-ODN or mixed with eND-CpG-ODN was 25 μg. The dose of CpG-ODN D19 bound to eND or M2ND was 10 μg. Fourteen days after the second vaccination, all the animals in the trial were challenged intranasally with 10^6^ PFU of H3N2 MN/16. Nasal swabs were collected at 0, 3 and 5 days after the virus challenge. Five days after the virus challenge, the animals were euthanized, their lungs harvested, and BALF collected. The titer of infectious virus in the nasal swabs collected at (A) 3 days and (B) 5 days after virus challenge, as well as (C) BALF was determined using a PFU assay. Each symbol represents the viral load for each pig. The horizontal bars represent the mean ± SE of the group. Data were analyzed by using the Kruskal-Wallis test. Asterisks denote significant differences with the eND-CpG-ODN vaccinated group: p <0.05, **p < 0.01, *** p <0.001.

**Fig. 11.**
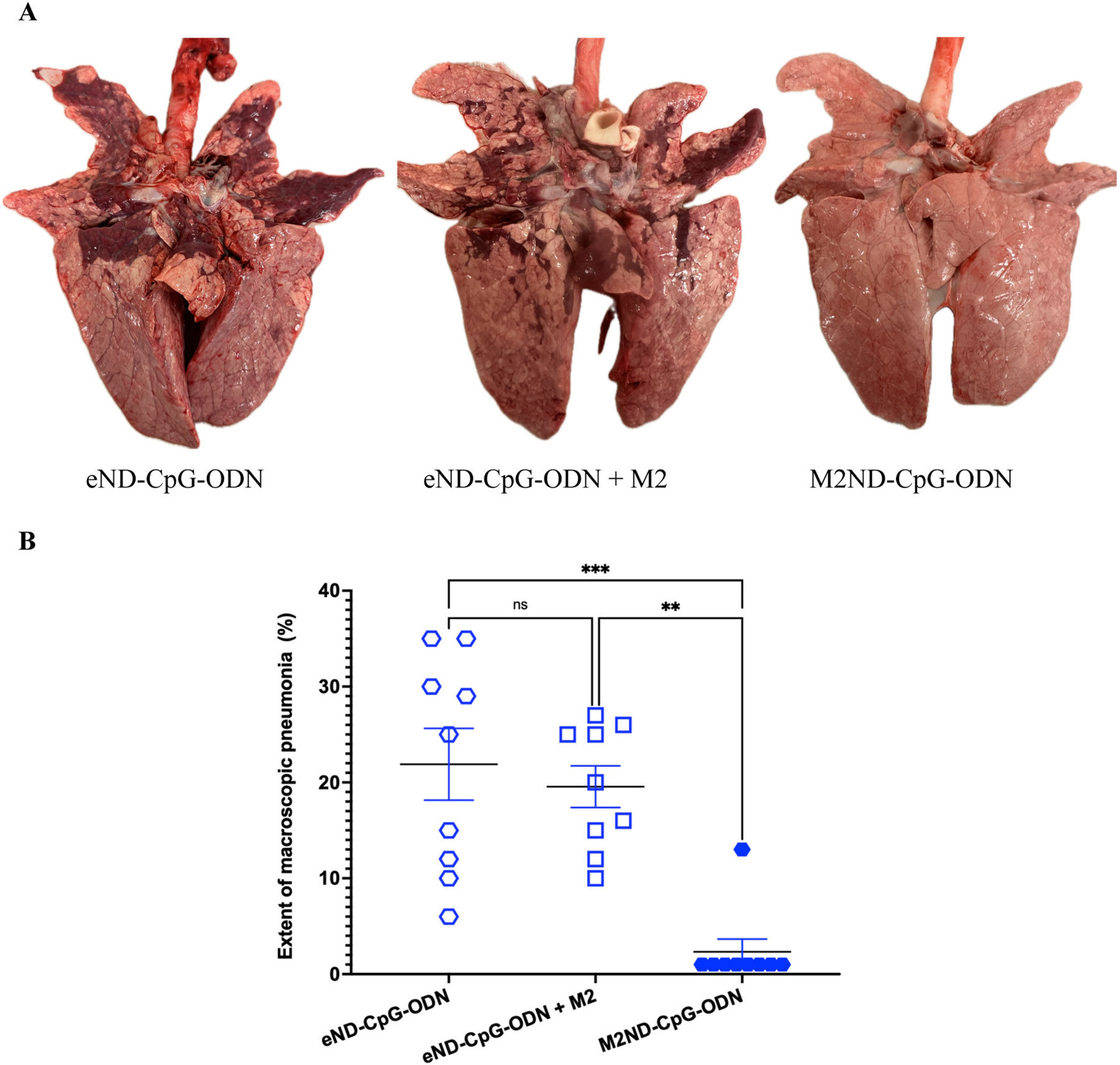
Extent of pneumonia in M2ND-CpG-ODN vaccinated pigs following a IAV challenge (Pig study 4). The same groups of pigs listed in Fig. 10 were euthanized 5 days after the virus challenge, their lungs removed and evaluated for the percentage of the lung affected with purple-red consolidation typical of IAV infection in swine. (A) Representative images of the ventral aspect of the lungs collected from pigs of the indicated group. (B) The percentage of the surface area affected by pneumonia was visually estimated for each lung lobe, and the total percentage of the entire lobe was calculated based on the weighted proportions of each lob to the total lung volume. Each symbol represents the extent of pneumonia for each pig. The horizontal bars represent the mean ± SE of the group. The gross pathology scores were analyzed using the Kruskal-Wallis test. Asterisks denote significant differences with the eND-CpG-ODN vaccinated group. ** p<0.01, *** p<0.001. ns: no significant difference.

The ability of M2ND-CpG-ODN to elicit protective immunity was tested by immunizing pigs both IM and IN twice at a 28-day interval with these nanostructures. Further, to ascertain the need for the M2 protein to be integrated into the ND structure for it to be an effective vaccine, a pig cohort was immunized with eND-CpG-ODN to which M2 was added by simply mixing with already formed NDs (eND-CpG-ODN + M2). The possibility that CpG-ODN could elicit non-specific protective immunity was appraised by including a pig cohort that was vacinated with eND that incorporated CpG-ODN but not M2 (eND-CpG-ODN). Accordingly, each group of pigs (n=9 each) was immunized with either: M2ND-CpG-ODN (group 1); eND-CpG-ODN + M2 (group 2); or eND-CpG-ODN (group 3) and challenged 17 days later with H3N2 MN/16. Before the challenge, none of the animals exhibited the presence of IAV in nasal swab samples (not shown). Except for a transient nasal discharge, challenge with either NY/11 or MN/16, even in mock-vaccinated pigs, did not induce any other clinical disease, such as sneezing, cough, lethargy, or loss of appetite (data not shown). Nasal swabs collected from animals in Group 1 at 3 (Fig. 10A) and 5 days (Fig. 10B) after virus challenge exhibited a >10-fold decrease of infectious virus as compared to animals in groups 2 and 3, which exhibited high titers of infectious IAV (p<0.01). Similarly, BALF collected from animals vaccinated with M2ND-CpG-ODN exhibited a significantly reduced amount of infectious virus 5 days after challenge (Fig. 10C) as compared to BALF obtained from the pigs in groups immunized with either eND-CpG-ODN (p<0.01) or eND-CpG-ODN + M2 (p<0.05). Notably, while the lungs of animals vaccinated with either eND-CpG-ODN + M2 (group 2) or eND-CpG-ODN (group 3) exhibited extensive pneumonia (Fig. 11), 8 of 9 pigs in the group vaccinated with the M2ND-CpG-ODN (group 1) exhibited the complete absence of pneumonic lesions in their lungs (p<0.01). Combined, these results indicate that the immunization of swine via both IM and IN routes with M2ND-CpG-ODN elicits significant levels of protective immunity against an IAV challenge.

## 4. Discussion

M2 is considered a promising target for the induction of protective immunity against IAV. Since this protein is very conserved, even between different subtypes of influenza A virus originating from various animal species, it is also considered as a vaccine candidate that could induce broadly protective humoral and cellular immune responses (Saelens et al., 2019). The M2 protein is present in the envelope of IAV at approximately 10–68 molecules per virion, which is about 10-fold less than the known number of HA molecules present in the virion’s membrane (Zebedde and Lamb, 1988). M2 is displayed as a homotetramer composed of two disulfide-linked dimers held together by non-covalent interactions that function as an ion channel, which is required for virus entry and egress (Sugrue and Hay, 1991; Holsinger and Lamb, 1991). The natural antibody response against M2, particularly against its ectodomain (M2e) in humans is very modest, likely due to the scarcity of M2 in the virus envelope (Feng et al., 2006; Deng et al., 2015). Similarly, in swine, a low antibody response to M2e was observed in the serum of animals after an experimental infection with IAV or vaccination with a WIV vaccine (Kitikoon et al., 2008). The high degree of M2 conservation combined with evidence that immunity against M2 can protect against a viral infection prompted major research efforts to explore the use of this molecule as a vaccine antigen to elicit broadly protective immunity (Mezhenskaya et al., 2019). Initial studies demonstrated that mice vaccinated with M2 produced in baculovirus (Slepushkin et al., 1995) or with DNA expressing a full-length consensus-sequence of M2 (Tompkins et al., 2007), elicited a strong antibody response and protected mice from a lethal challenge with a M2 matched IAV. The immunization of swine with M2 produced in baculovirus was found to induce a low-level production of M2e-specific antibodies, but it was not protective (Kitikoon et al., 2009). In contrast, our results show that IM immunization of swine with M2NDs elicits the development of a strong M2 and M2e specific antibody response that was >7-fold higher than the response elicited against these two antigens after immunization with a commercial inactivated influenza virus vaccine. Importantly, the antibody response elicited by the M2NDs reacted with influenza virions, indicating that the antibodies generated recognize the structure of M2 as it is naturally expressed on influenza virions. Similarly, IN immunization with M2NDs stimulated the production of IgA in BALF that reacted with M2, M2e and, most importantly, influenza virions.

Although the entire M2 protein is highly conserved, the N-terminal 23 amino acids, which protrude from the viral envelope and represent M2e, are the most conserved (Deng et al., 2015). The high sequence conservation of M2e was central to it becoming a focus antigen for the development of a universal human influenza A vaccine. However, because M2e is poorly immunogenic, it is often fused with a larger carrier to enhance anti-M2e immune responses in vaccination experiments (Schepens et al., 2018). Genetic fusion of M2e to the hepatitis B virus core (HBc) protein expressed in *E. Coli* generated a M2eHBc fusion protein which forms a virus-like particle (VLP) with M2e radiating from its surface. Intraperitoneal or IN immunization of mice with M2eHBc was shown to elicit the generation of M2e-specific antibodies as well as confer immune protection against a lethal virus challenge (Neirynck et al., 1999; De Filette et al., 2005). Notably, no protective immunity was observed when the M2eHBc, created by Neirynck et al., was tested for its efficacy in swine (Heinen et al., 2002). However, the sequence of the M2e in the M2eHBc vaccine was derived from a consensus sequence of M2e from human influenza A viruses which differed in 6 of the 23 amino acids from the M2e sequence present in the European swine influenza virus used to challenge the pigs. Another study in swine, which tested two experimental M2e-based subunit vaccines, similarly failed to show evidence of protective immunity against a challenge with IAV. In this case, the two M2e-based subunit vaccines were prepared using the amino acid sequence of the triple-reassortant internal gene (trig) isoform of M2 (or a close variant of it), and the animals were challenged with a IAV carrying the pM gene segment (Opriessnig et al., 2018). Accordingly, the M2 expressed by the challenge virus differed from the M2e in the vaccines by 5 amino acids in one case, and by 4 in the other. The failure of the M2e-based vaccines to protect swine in these two reports highlight the need to have a close to perfect match between the 23 amino acid long sequence of a M2e-based vaccine and the challenge virus. In contrast, by taking into consideration the need for a close match between the vaccine and the challenge virus, several types of VLPs displaying M2e have been successfully tested in animal models (mostly in mice) for vaccine efficacy against a panel of divergent IAV (Deng et al., 2015). As a result, some M2e vaccine candidates have advanced to Phase I and Phase II human clinical stage trials (Kolpe et al., 2017). It has been recommended that the design of a M2e-based human vaccine include four different M2e consensus sequences to protect against all plausible IAV subtypes circulating in both human and animal reservoirs. This strategy would maximize the cross-reactivity of the antibody response to M2e against all circulating human, avian, and swine influenza viruses that could affect the human population (Mezhenskaya et al., 2019). Currently >99 % of the IAV in swine passively monitored through a U.S. Department of Agriculture (USDA) administered surveillance system express the pM gene segment (Arendsee et al., 2021). Therefore, a single M2 sequence would be sufficient to create a M2-based vaccine to induce immunity against >99 % of IAV strains afflicting commercial swine, at least in the USA.

NDs are well defined nanostructures used extensively to display membrane proteins (Bayburt et al., 2002; Luthra et al., 2013; Denisov and Sligar, 2017; Sligar et al., 2021). Because NDs have been used to deliver IAV HA as a vaccine antigen capable of eliciting protective immunity (Bhattacharya et al., 2010), we chose to use this type of nanostructure to deliver the entire M2 as a vaccine antigen. Given that expanding the breadth of immunity afforded by IAV WIV vaccines to swine is a desirable goal (Vincent et al., 2017), our initial efforts were aimed at exploring the ability of the M2ND to enhance the protective immunity afforded by a conventional WIV vaccine, which under HA mismatched conditions are known to only provide partial protective immunity (Loving et al., 2013). We chose to use two HA antigenically distinct strains of H3N2 Clade IV viruses that differ in their H3 antigenic motif. While the H3 antigenic motif expressed by the NY/11 belongs to the red antigenic cluster, the one expressed by the MN/16 belongs to the green antigenic cluster (Bolton et al., 2019). This combination of H3 antigenically mismatch between vaccine and challenge virus has been shown to only provide partial protection (Abente et al., 2018; Abente et al., 2019). Fittingly, our M2ND vaccine was designed using the pdm isoform of M2, and the animals were challenged with viruses expressing the same M2 isoform. The results of pig study 2 showed that concurrent immunization with the WIV vaccine and M2ND increased the protective efficacy against the H3 mismatched challenge virus as demonstrated by a diminished viral load in the lower respiratory tract. However, this strategy did not increase the rate of elimination of the virus from nasal secretions nor did it increase the ability of the WIV vaccine to lessen the emergence of pneumonia resulting from the virus challenge. In the same study, evidence that immunization with M2ND as a stand-alone biologic can stimulate protective immunity was provided by the elimination of the virus from the BALF in two pigs and a >10-fold reduction of the viral load in three others. This observation encouraged us to perform an additional experiment designed to assess the protective efficacy of M2ND vaccine using an equal number of pigs in the vaccinated and the placebo groups to maximize statistical power. We also changed the type of adjuvant to formulate the M2ND vaccine to a different type of emulsion adjuvant that we (Pig study 2) and others (Vincent et al., 2010; Abente et al., 2018) have used to prepare effective WIV vaccines. Notably, the results of Pig study 3 were remarkably like those observed in Pig study 2, namely in both cases more than half of the pigs in the M2ND vaccinated group exhibited either a >10-fold reduction or complete elimination of the challenge virus from the BALF. However, in both studies, IM immunization with M2ND mixed with an emulsion adjuvant did not diminish the viral load in nasal secretion nor the extent of pneumonia that ensued after the virus challenge.

In pigs (Martini et al., 2021), as well as in mice (Tateishi et al., 2019), concurrent systemic and intranasal immunization is an effective strategy to induce protective immunity against IAV in both the upper and lower respiratory tracts. Hence, we used this mode of a dual route of vaccination to further test the efficacy of M2NDs. We also modified the formulation of the M2ND biologic by incorporating CpG-ODN into the nanostructure, which has strong vaccine adjuvant properties (Kayraklioglu et al., 2021) and works best when they are incorporated directly into the nanostructure displaying the antigen (Fischer et al., 2013; Kuai et al., 2017). Our results revealed that immunization of pigs with the M2ND-CpG-ODN vaccine alone decreased the viral load after the virus challenge in both the upper and lower respiratory tracts and fully eliminated the occurrence of lung pathology in eight of the nine pigs in the group vaccinated with this biologic. Notably, immunization with M2 not embedded in the ND structure (i.e., the eND-CpG-ODN + M2 vaccinated group) failed to elicit protective immunity. This observation indicates that for the M2 to function as an effective vaccine, it must be displayed in its transmembrane configuration in a lipid bilayer. This observation is consistent with the report that IM immunization of swine with M2 produced in baculovirus neither reduced virus shedding, nor did it significantly reduce the extent of pneumonia (Kitikoon et al., 2010). As a stand-alone biologic, immunization with the M2ND-CpG-ODNs did not diminish or eliminate the challenge virus from nasal secretions as effectively as the monovalent WIV vaccine did, even in the H3 mismatched challenge. However, given the observed ability of the M2ND-CpG-ODN vaccine to uniformly reduce the viral load in nasal secretions in all vaccinated pigs in the group; to reduce or eliminate the virus from BALF; as well as to impede the emergence of pneumonia, it is apparent that a M2ND-CpG-ODN vaccine is a promising approach for overcoming the limitation of strain-specific protection afforded by commercially available WIV vaccines. This notion is supported by the report that in mice, supplementation of a H1N1 WIV vaccine with M2 VLPs significantly improved cross protective efficacy against a lethal challenge with heterologous IAV, including the 2009 H1N1 pandemic virus, as well as heterosubtypic H3N2 and H5N1 IAV (Song et al., 2011).

Protective immunity against IAV infection involves both humoral and cell mediated immunity (McMichael et al., 1983; Hillaire et al., 2011; El Bakkouri et al., 2011; Sridhar et al., 2013; Altenburg et al., 2015; Lee et al., 2015; Morgan et al., 2016; Sant et al., 2018; Lee et al., 2019). In swine, following an experimental infection with IAV, virus clearance is associated with the emergence of IAV-specific T and B cell immunity including the presence of IAV-specific CD8 T cells producing both IFN-γ and tumor necrosis factor (TNF) in the airways, as well as neutralizing antibodies in serum and lung fluids (Edmans et al., 2021). Although IAV-specific neutralizing antibodies are considered a principal mechanism of defense against IAV, non-neutralizing antibodies are likely to be involved in virus clearance and protection via Fc domain effector functions (Ma et al., 2023). The ability of several porcine IgG subclasses to perform Fc-mediated effector functions against IAV including complement mediated cellular cytotoxicity (CDCC), antibody dependent cellular cytotoxicity (ADCC), and antibody mediated cell phagocytosis (ADCP) was recently described (Paudyal et al., 2022). Notably, antibodies specific against M2e have been shown to mediate protective immunity against IAV via all three Fc-mediated effector functions listed above (reviewed in Lee et al., 2015). Our studies show that immunization with M2ND either elicited the production of IgG capable of reacting with M2e, M2 and, most importantly, influenza virus. Determining the effector function capabilities of these porcine M2 specific antibodies will be the subject of future studies. Regarding the role of M2-specific T cells in mediating protective immunity, M2e-specific CD4+ and CD8+ T cells have been shown to contribute to improving heterosubtypic cross protection in mice (Kim et al., 2014). However, there are several T cell epitopes present in the transmembrane and intracellular domains of M2 (Stoloff and Caparros-Wanderley, 2007; Deng et al., 2015; Tutykhina et al., 2018). By only using M2e as the vaccine antigen, the potential to elicit cell-mediated immunity to the T-cell epitopes known to be present in the transmembrane and intracellular domains of M2 is not employed. Hence, missing the opportunity to utilize the full potential of this conserved protein to provide protective cell-mediated immunity. Indeed, the novel peptide vaccine candidate FLU-v, which is designed to elicit a broad-spectrum T-cell response specific for conserved viral antigens, includes (as one of the four peptides constituting this vaccine) a highly conserved 24 amino acid long peptide located in the transmembrane domain of M2 (Oftung et al., 2022). The potential that cross-reactive memory T cells specific for conserved IAV proteins, such as the nucleoprotein, can have in mediating protective immunity against influenza in swine was made evident by their presence in the lungs of swine infected with IAV (Talker et al., 2016). Our approach to use NDs to deliver M2 in its entirety makes it possible to elicit cell-mediated immunity to all the potential epitopes present in the M2 protein. The observation that the frequency of IFN-γ-SC detected in response to the stimulation with M2 of PBMC from M2ND vaccinated pigs was >2 fold higher than the frequency detected in response to stimulation of the same cell population with M2e suggests that one or more T-cell epitope besides the M2e T cell epitope were being recognized.

From the population medicine point of view, an IAV for swine vaccine should reduce the transmission of the virus within the population. Commercially available IAV vaccines for swine are primarily adjuvanted WIV vaccines delivered IM, which offer limited efficacy against infection and transmission by heterosubtypic IAV strains (reviewed in Ma et al., 2023). Since a reduced rate of virus shedding can delay the spreads of the virus (Loving et al., 2013), it is plausible that the infection-permissive immunity provided by the M2ND-CpG-ODN as a supplement to the immunity provided by WIV under HA mismatched conditions might sufficiently reduce the virus transmission and help control virus spread. This important aspect of vaccine efficacy remains to be assessed. Although we have not yet tested the efficacy of the M2ND-CpG-ODN against a different IAV subtype, it is reasonable to expect that protection can be conferred, provided that the M2 isoforms of the vaccine and challenge virus are matched. Perhaps the most important outcome of this study is the demonstration that an M2-based vaccine confers protective immunity in a species that is the natural host for IAV, as well as the target species for this biologic. Given that swine is considered a good model for IAV research (Meurens et al., 2012; Margine and Krammer, 2014; McNee et al., 2020; Nguyen et al., 2021), this study encourages the development of M2-based vaccines for human use, and points to the value of using swine to test the efficacy of novel biologics against IAV. In conclusion, this study demonstrates that the immunization of swine with the entire M2 displayed in NDs provides significant levels of protective immunity against IAV. We are currently striving to further improve the formulation of the M2ND-CpG-ODN vaccine to maximize its efficacy so that it can optimally function either as a potent stand-alone biologic or as a supplementary immunogen to commercially available IAV WIV vaccines to help overcome their limitation of strain-specific protection. The M2ND vaccine offers great promise as a supplement to WIV vaccines or as a broadly protective IAV vaccine to solve the challenges presented by the great diversity of IAV present in the field and help reduce the incidence and spread of IAV in swine.

## Figures and Tables

**Table 1:** Experimental design of pig studies.

Pig study	Treatment group	Pigs per group	Vaccine - adjuvant (route)	Challenge virus (route)	Data location
**1**	1	4	M2ND - ISA206 (IM)	None	Figs. 2 and 3
	2	4	M2ND - *M. smegmatis* (IN)		
	3	4	FluSure XP - Amphigen^^®^^ (IM)		
	4	4	eNDs - ISA206 (IM) + eNDs - *M. smegmatis* (IN)		
**2**	1	8	WIV NY/11 H3N2 - Emulsigen-D (IM) + M2ND - ISA206 (IM)	MN/16 H3N2 (IT)	Figs. 4 and 5
	2 A	8	WIV NY/11 H3N2 - Emulsigen-D (IM)		
	3	8	M2ND - ISA206 (IM)		
	4	4	Vehicle (saline) - Emulsigen-D (IM) + eND - ISA206 (IM)		
	2B	5	WIV NY/11 H3N2 - Emulsigen-D (IM)	NY/11 H3N2 (IT)	
	5	4	Vehicle (saline) - Emulsigen-D (IM)		
	6	4	Strict control	None	
**3**	1	7	M2ND - Emulsigen-D (IM)	MN/16 H3N2 (IT)	Figs. 6–8
	2	7	eND - Emulsigen-D (IM)		
	3	2	Diluent (saline) Emulsigen-D (IM)		
	4	2	Strict control	None	
**4**	1	9	M2ND-CpG-ODN (IN/IM)	MN/16 H3N2 (IN)	Figs. 9–11
	2	9	eND-CpG-ODN + M2 (IN/IM)		
	3	9	eND-CpG-ODN (IN/IM)		
